# Residues in the fructose-binding pocket are required for ketohexokinase-A activity

**DOI:** 10.1016/j.jbc.2024.107538

**Published:** 2024-07-04

**Authors:** Juliana C. Ferreira, Adrian J. Villanueva, Samar Fadl, Kenana Al Adem, Zeynep Nur Cinviz, Lyudmila Nedyalkova, Thyago H.S. Cardoso, Mario Edson Andrade, Nitin K. Saksena, Ozge Sensoy, Wael M. Rabeh

**Affiliations:** 1 Science Division, New York University Abu Dhabi, Abu Dhabi, United Arab Emirates; 2 Graduate School of Engineering and Natural Sciences, Istanbul Medipol University, Istanbul, Turkey; 3 Structural Genomics Consortium, University of Toronto, Toronto, Ontario, Canada; 4 G42 Healthcare Omics Excellence Center, Abu Dhabi, United Arab Emirates; 5 Horticultural Sciences Department, University of Florida, Gainesville, Florida, USA; 6 Institute for Health and Sport, Victoria University, Melbourne, Victoria, Australia; 7 Regenerative and Restorative Medicine Research Center (REMER), Research Institute for Health Sciences and Technologies (SABITA), Istanbul Medipol University, Istanbul, Turkey

**Keywords:** ketohexokinase, fructokinase, metabolic syndrome, cancer, obesity, thermodynamic stability, molecular dynamics, enzyme kinetics

## Abstract

Excessive fructose consumption is a primary contributor to the global surges in obesity, cancer, and metabolic syndrome. Fructolysis is not robustly regulated and is initiated by ketohexokinase (KHK). In this study, we determined the crystal structure of KHK-A, one of two human isozymes of KHK, in the apo-state at 1.85 Å resolution, and we investigated the roles of residues in the fructose-binding pocket by mutational analysis. Introducing alanine at D15, N42, or N45 inactivated KHK-A, whereas mutating R141 or K174 reduced activity and thermodynamic stability. Kinetic studies revealed that the R141A and K174A mutations reduced fructose affinity by 2- to 4-fold compared to WT KHK-A, without affecting ATP affinity. Molecular dynamics simulations provided mechanistic insights into the potential roles of the mutated residues in ligand coordination and the maintenance of an open state in one monomer and a closed state in the other. Protein–protein interactome analysis indicated distinct expression patterns and downregulation of partner proteins in different tumor tissues, warranting a reevaluation of KHK’s role in cancer development and progression. The connections between different cancer genes and the KHK signaling pathway suggest that KHK is a potential target for preventing cancer metastasis. This study enhances our understanding of KHK-A's structure and function and offers valuable insights into potential targets for developing treatments for obesity, cancer, and metabolic syndrome.

The growing prevalence of obesity is now recognized as a global pandemic (1, 2). Obesity is a complex condition attributed to an array of sociodemographic, lifestyle, genetic, and environmental factors (3, 4, 5). However, a large body of experimental, epidemiological, and clinical evidence implicates the consumption of foods with high fructose contents as a significant contributing factor (6, 7, 8, 9, 10, 11, 12). Fructose is a common monosaccharide found in table sugar, high-fructose corn syrup, and fruit (13), and its consumption has increased continuously since the early 20th century (14, 15). Fructose consumption may also be the main cause of metabolic syndrome (MetS), a set of metabolic abnormalities that includes hypertension, insulin resistance, fatty liver disease, and atherogenic dyslipidemia (15, 16, 17, 18, 19).

At the molecular level, the negative effects of fructose on health are often ascribed to the lack of regulation of the fructose metabolic pathway. Unlike glucose metabolism, fructose catabolism bypasses phosphofructokinase (PFK), a vital glycolytic checkpoint (12). Instead, the breakdown of fructose is initiated by ketohexokinase (KHK), also known as fructokinase. KHK catalyzes the phosphorylation of the C1 hydroxyl of fructose to generate fructose-1-phosphate (F-1P) (20, 21). The activity of KHK is not regulated by ATP or intermediate products in the downstream fructolytic pathway (22). As a result, hepatic uptake differs greatly between glucose and fructose. The *K*_m_ of hepatic glucokinase for glucose is high, enabling the body to establish balanced blood sugar levels (23). By contrast, unregulated KHK has high substrate affinity, effectively preventing the bulk of fructose from entering the bloodstream (24). Ultimately, fructose metabolism joins glucose metabolism at the levels of glyceraldehyde 3-phosphate and dihydroxyacetone phosphate, continuing to complete glycolysis and pyruvate production (24). Collectively, the divergence of fructose catabolism from the glycolytic pathway and the lack of control of KHK activity lead to poorly regulated fructose metabolism.

KHK is most abundant in the liver, but low levels of KHK are also found in the kidney, intestine, brain, pancreas, lung, muscle, and optic nerve (25, 26, 27, 28). Alternative splicing results in the production of two human KHK isozymes: KHK-A and KHK-C. In most cells, the expression of these isozymes is mutually exclusive (29). In mice, KHK-C is found in the liver (the principal site of fructose metabolism) and kidney, whereas KHK-A is ubiquitously expressed in other cells (30). In addition to location, KHK isoforms differ significantly in their kinetic parameters. The affinity of hepatic KHK-C for fructose is much higher than that of KHK-A (31), and KHK-C is consequently considered the primary player in fructose metabolism. However, KHK-A has been implicated in tumor development. Hepatocellular carcinoma tumor cells switch the splicing of KHK-C to KHK-A, which activates phosphoribosyl pyrophosphate synthetase 1 to drive nucleic acid synthesis for tumor development (1, 32). Because KHK is the first step in fructolysis, biochemical and structural characterization of its isozymes is important for addressing knowledge gaps and opening potential new avenues to effective therapies for the chronic effects of obesity, cancer, and MetS.

KHK is a member of the phosphofructokinase B (pfkB) family, one of three distinct families that encompass all known carbohydrate/purine kinases. Kinases in the pfkB family, which also includes ribokinase (RK) and adenosine kinase (33, 34, 35), are characterized by two structural elements: a large central α/β domain where the active site is located and a small lid domain that covers it upon substrate binding and catalysis (36). Although members of the pfkB family have weak sequence identities, their crystal structures have revealed homologous tertiary structures with a core Rossman-like fold (37, 38, 39). Furthermore, pfkB family members contain two conserved sequences: at the N terminus, a glycine-rich area with two consecutive glycine residues functions as the hinge between the α/β central and lid domains (36); in the C-terminal region, the second conserved sequence is involved in ATP binding and contains an aspartate residue that serves as the catalytic residue for initiating fructose phosphorylation. Although a catalytic mechanism for KHK has yet to be proposed, the crystal structures of human KHK-A and KHK-C indicate that Asp258 acts as a general base according to the mechanism suggested for RK (37), abstracting a proton from the C1 hydroxyl of fructose to enable nucleophilic attack of the oxygen on the γ-phosphate of ATP, producing F-1P and ADP (34). It has been shown previously and in the work presented here that the crystal structure of KHK-A in complex with both substrates reveals similar conformations upon binding the substrates with minor differences at the loop after the last β-strand of the β-clasp (37). However, a thorough biochemical and biophysical analysis of KHK is imperative to unravel its structural attributes and catalytic mechanisms.

In this study, we determined the crystal structure of human KHK-A in the apo-state at a high resolution of 1.85 Å (Protein Data Bank (PDB) code: 2HLZ). Furthermore, multiple mutations were introduced in the fructose-binding pocket of KHK-A to map the active site and characterize the roles of these residues in fructose binding, catalysis, and overall protein stability. The mutants were kinetically and thermodynamically characterized by performing initial velocity studies, differential scanning calorimetry (DSC), and mass photometry (MP). Additionally, atomistic molecular dynamics (MD) simulations were used to investigate differences in the structural fold and conformational rearrangements of the WT and mutant enzymes during catalysis. The results provide new insights into the important roles of the fructose-binding pocket residues in the catalytic mechanism and structural stability of KHK-A.

## Results

### Structural analysis of human KHK-A

KHK-A was expressed in *Escherichia coli* and purified by Ni-NTA affinity and size-exclusion chromatography. The final protein had a purity of >95% according to Coomassie staining SDS–PAGE analysis (data not shown). The crystal structure of apo-human KHK-A was determined to 1.85 Å resolution using crystals grown at 291 K and pH 4.2 by the sitting-drop vapor-diffusion method (PDB code: 2HLZ). The final structure contained two homodimers in the asymmetric unit. Each KHK-A monomer was well refined, with good electron density for residues 3 to 298, a crystallographic R-factor of 0.216, and an R-free value of 0.249 (Table S1).

**Figure 1:**
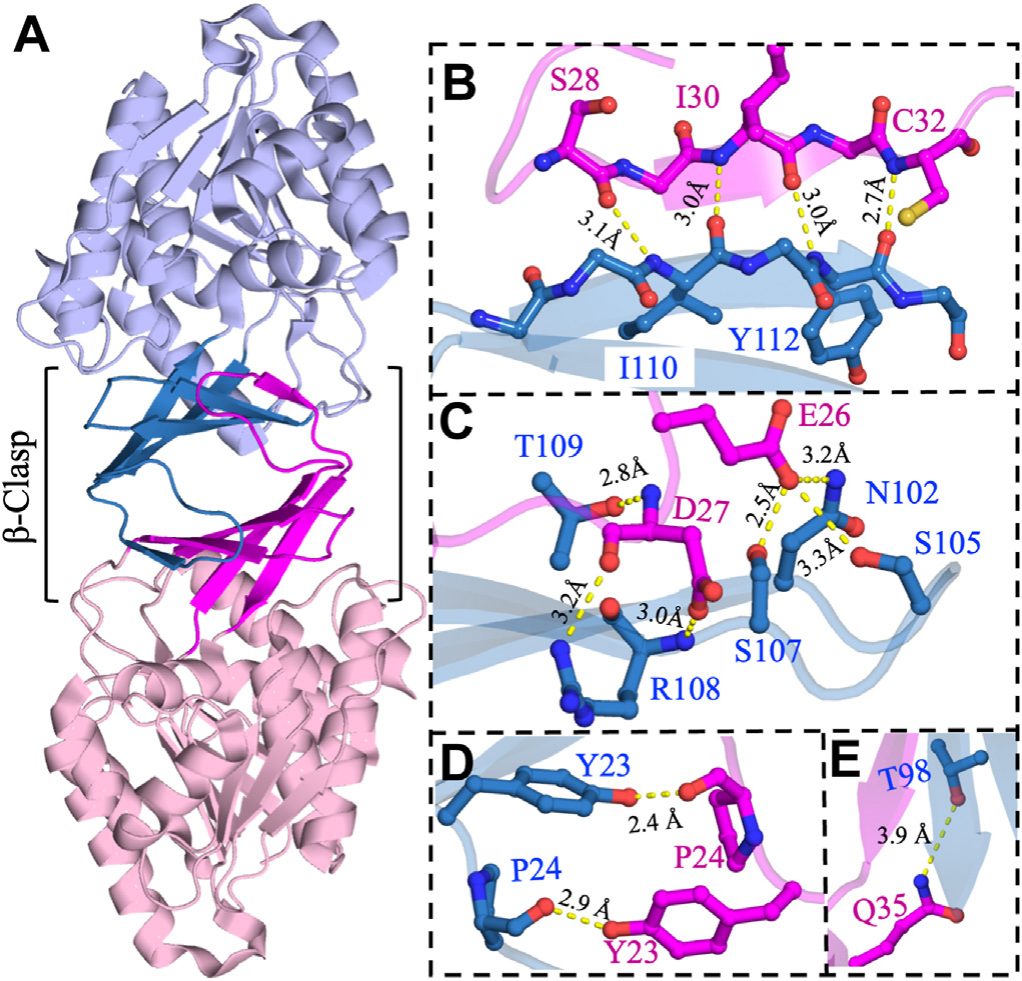
**Structural analysis of the dimer interface of KHK-A.***A*, cartoon representation of the crystal structure of the homodimer of human KHK-A (PDB: 2HLZ). One monomer is shown in *blue*, and the other monomer is shown in *pink*. The dimer interface exhibits a spatial arrangement called the β-clasp, which is formed through interactions of the extended four-stranded β-sheets of each monomer. Residues 21 to 31 are important for the dimerization of KHK-A, as they form strand β3a and the loop region between strands β2 and β3a. *B*, multiple interdomain interactions are formed in the β-clasp structure, including hydrogen-bonding interactions between strand β3a of one monomer and strand β7 of the other monomer. Strand β3a forms the fifth strand of the four-stranded β-sheet of the other monomer, which has the same hydrogen-bonding patterns as a parallel β-sheet. *C*, Loop^20–29^ between strands β2 and β3a forms bonding interactions with loop^102–108^ between strands β6 and β7 of the other monomer. E26 may form hydrogen bonds with N102, S105, and S107 of the other monomer. In addition, the side chains of R108 and T109 of one monomer interact with the backbone of D27 of the other monomer. *D* and *E*, the center of the β-clasp structure features two contact points between the monomers, as Y23 of one monomer forms hydrogen bonds with the backbone of P24 of the other monomer. Additionally, the side chains of Q35 form hydrogen bonds with T98 of the other monomer. This unique set of interactions is the principal characteristic of the β-clasp structure found in the pfkB family. The figure was produced using PyMOL. KHK, ketohexokinase; PfkB, phosphofructokinase B; PDB, Protein Data Bank.

Each KHK-A monomer consists of two domains. The central α/β domain comprises a nine-stranded β-sheet flanked on each side by five α-helices, resembling the conserved Rossman-like fold (40) (Fig. S1*A*). A four-stranded β-sheet extends from the central α/β fold to form the lid domain. Residues 13 to 40 (β2 and β3) and 96 to 113 (β6 and β7) form a four-stranded antiparallel β-sheet that is part of the lid domain (Fig. S1*B*). The protruding β-sheets of the lid domain of each monomer interact with each other to form the dimer interface of KHK-A. The two monomers are related by a two-fold axis of rotational symmetry, forming an isologous interface (41, 42). In other words, two pairs of identical interactions stabilize the dimer interface. The protruding β-sheets of each monomer are packed and oriented orthogonally to each other (43). Additionally, a central bend in strand β3 wraps around and interacts with strand β7 of the other monomer. The combination of these structural features produces a unique arrangement referred to as the β-clasp structure, a spatial configuration that resembles a “handshake” (Fig. 1*A*) (34, 37).

Residues V20–S34, which connect strands β2 and β3b, form long loop^20–29^ and strand β3a (Fig. S2). Loop^20–29^ and strand β3a extend to reach the other monomer at its four-stranded β-sheet lid domain, interacting with residues on strand β7 and loop^102–108^ between strands β6 and β7 (Fig. 1, *A* and *B*). Interestingly, in the β-clasp, the backbone strand β3a of one monomer forms hydrogen bonding interactions with the strand β7 of the four-stranded β-sheet of the other monomer (Fig. S1). For example, S28, I30, and C32 of strand β3a stabilize the dimer interface by forming backbone–backbone hydrogen bonding interactions with residues I110 and Y112 of strand β7 of the other monomer (Fig. 1*B*). In particular, the backbone carbonyl oxygen of S28 interacts with the backbone nitrogen of I110 at 3.1 Å. In addition, the backbone carbonyl oxygen and nitrogen of I30 form hydrogen bonds with the backbone carbonyl oxygen of I110 and nitrogen of Y112 at 3.0 Å (Fig. 1*B*). Finally, the backbone nitrogen of C32 forms a hydrogen bond with the backbone carbonyl oxygen of Y112 at 2.7 Å. This set of hydrogen bonding interactions is typical of parallel β-sheets and is the principal characteristic of the β-clasp structure (34, 37, 44).

**Figure 2:**
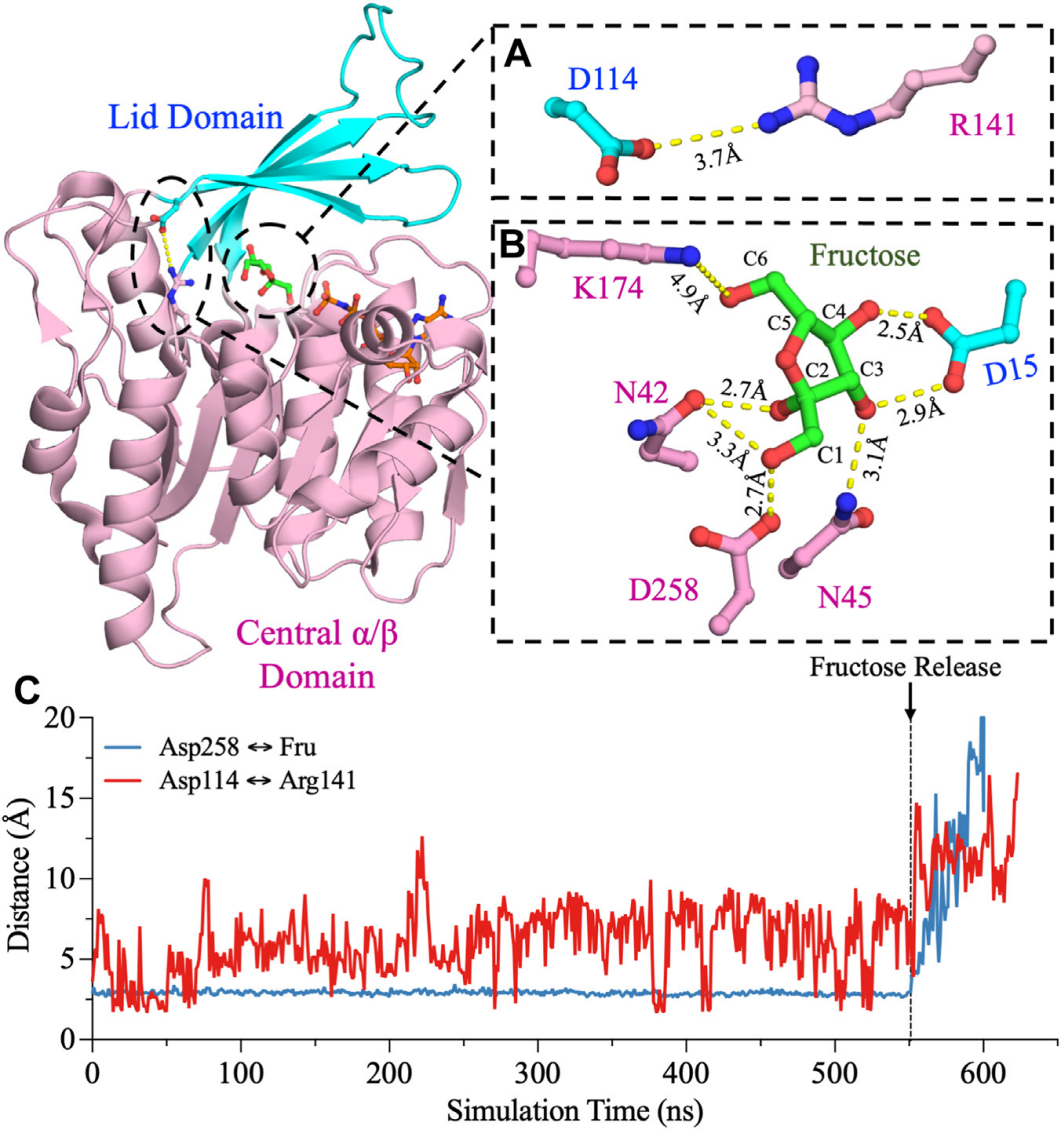
**The crystal structure of human KHK-A in complex with fructose and AMP-PNP (PDB:****2HW1****).** Fructose is bound to the active site, which is located at the cleft between the central α/β domain and the lid domain. *A*, the side chain of D114 of the lid domain forms an ionic interaction at 3.7 Å with the side chain of R141 of the central α/β domain. These interactions may be important for the opening and closing mechanism of KHK-A, and they are >5 Å away from the fructose binding site. *B*, fructose makes polar interactions with the catalytic residue, D258, and other residues in the active site, such as D15, N42, N45, and K174. The figure was produced using PyMOL. *C*, comparison of the distances between D258 and fructose and between D114 and R141. The distances between both pairs of partners increase upon fructose release. KHK, ketohexokinase; PDB, Protein Data Bank.

Additional interdomain interactions in the β-clasp structure include those between loop^20–29^ and loop^102–108^. The side chain of E26 forms hydrogen bonding interactions with the side chains of N102, S105, and S107 of the other monomer at 3.2 Å, 3.3 Å, and 2.5 Å, respectively (Fig. 1*C*). In turn, D27 and R108 engage in backbone interactions between monomers: the side chain of D27 forms a hydrogen bond with the backbone nitrogen of R108 of the other monomer at 3.0 Å, and the side chain of R108 forms a hydrogen bond with the backbone carbonyl oxygen of D27 of the other monomer at 3.2 Å (Fig. 1*C*). Additional interdomain interactions in the β-clasp are at the core of the dimer interface; the side chain of Y23 hydrogen bonds the backbone carbonyl oxygen of P24 of the other monomer at 2.4 Å or 2.9 Å (Fig. 1*D*). Furthermore, the side chain of T109 hydrogen bonds the backbone nitrogen of D27 at 2.8 Å at the center of the dimer interface (Fig. 1*E*). In the lid domain, D114 extends out of the last β-strand of the β-clasp domain to form side-chain interactions at 3.7 Å with R141 of the central α/β domain (Fig. 2*A*).

The previously resolved crystal structure of KHK-A in complex with both substrates—fructose and AMP-ANP, an analog of ATP (PDB: 2HW1)—shows that the active site is located at the cleft between the central α/β fold and the lid domains (Fig. 2) (37). Fructose participates in several polar interactions with the active-site residues of KHK-A. The C1 hydroxyl of fructose forms hydrogen bonds with the side chain of the catalytic residue, D258, at 2.7 Å and the side chain of N42 at 3.3 Å (Fig. 2*B*). In addition, the C2 hydroxyl forms a hydrogen bond with the side chain of N42 at 2.7 Å, and the C3 hydroxyl forms two possible hydrogen bonds with the side chains of D15 (2.9 Å) and N45 (3.1 Å). The C4 and C6 hydroxyls form hydrogen bonds with the side chains of D15 and K174 at 2.5 Å and 4.2 Å, respectively.

**Figure 3:**
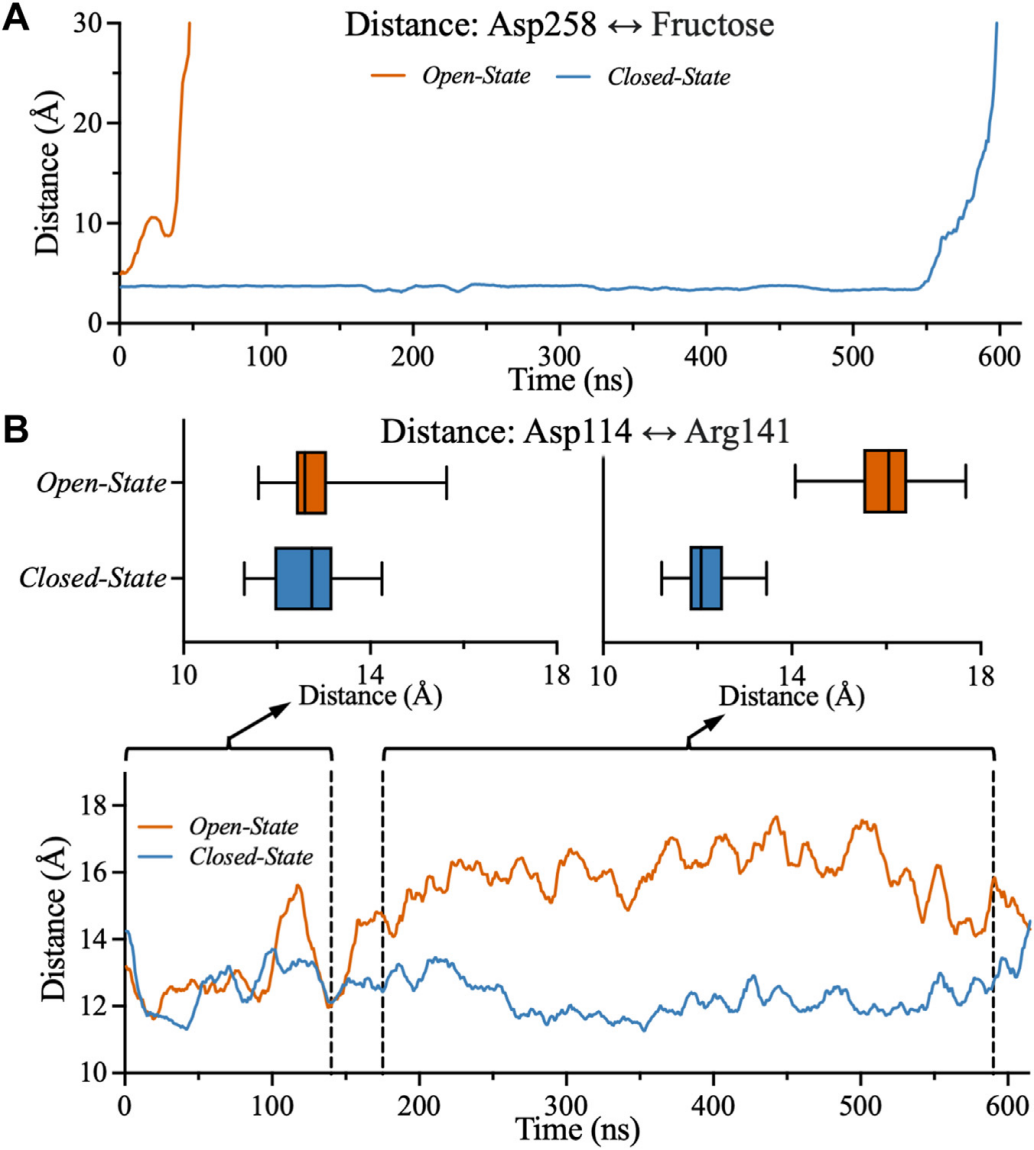
**Investigation of the occupancy states of dimeric WT KHK-A.** The molecular distances between the protomers in the closed and open states were obtained from MD simulations of fructose binding in WT KHK-A. *A*, the distance between D258 and fructose shows that fructose remains inside the binding pocket in the closed state of KHK-A but far from the binding pocket in the open state of KHK-A. *B*, from 0 ns to 140 ns, the distance between D114 and R141 does not change. However, from 175 ns to 590 ns, the distance in the open state begins to increase, while the distance in the closed state decreases slightly. These distances were calculated by comparing the distances between the C_α_ carbons of the corresponding residues. KHK, ketohexokinase; MD, molecular dynamics.

To further characterize the binding pockets of KHK, atomistic MD simulations were performed using a model based on the crystal structure of human apo-KHK-A (PDB: 2HLZ) in which ATP and fructose were introduced into the KHK-A active site. The fructose affinity of each protomer of the homodimer was estimated from the rate of fructose departure, which was monitored by measuring the distance between the catalytic residue D258 and the C1 hydroxyl of fructose (Fig. 2*C*). The rate of fructose departure was further confirmed by the distance between K174 of the active site and the C6 hydroxyl of fructose, the nearest hydroxyl group (Fig. S3*A*). The MD trajectories of the KHK-A dimer revealed a unique binding mode between the two protomers. Fructose left one protomer almost immediately after the onset of the simulation, whereas the other protomer retained fructose in its binding pocket 10 times longer (Fig. 3*A*). The protomer that released fructose early was classified as being in an open state, whereas the protomer that retained fructose longer was classified as being in a closed state. These trajectories support a catalytic mechanism involving conformational changes in which one protomer opens while the other closes.

To further correlate the rate of fructose release with the opening and closing of KHK-A, the distance between the C_α_ of D114 and R141 pair was used to monitor the opening and closing mechanism of KHK-A’s active site during MD simulations. The distance of 3.7 Å between R141 and D114 can be used to represent the distance between the central α/β fold and lid domains of KHK (Fig. 2*A*). Therefore, the R141 interactions with D114 can be used to demonstrate the opening/closing of the binding pocket of KHK throughout the MD simulations. This distance increased by more than 10 Å, when the distance between the D258 and fructose increased by more than 2.7 Å (Fig. 2*C*). When the protomer in the open state unfurled at approximately 175 ns, the distance between the C_α_ of D114 and R141 increased from 12 Å to 16 Å (Fig. 3*B*), whereas these residues maintained a fixed distance of 12 Å in the protomer in the closed state. These observations further indicate that when one protomer opens, the other stays in the closed state. Similarly, the distance between the D144 and K174 pair increased in the protomer in the open state after 200 ns compared to the protomer in the closed state (Fig. S3*B*). This conformational cooperativity could result from the dual role of the β-clasp, which covers the active site upon substrate binding and stabilizes the dimer interface. Upon substrate entry or exit, the lid domain of one protomer opens, pushing against the lid domain of the other protomer and maintaining it in the closed state.

### Effects of mutations in the fructose-binding pocket on activity and oligomerization

**Figure 4:**
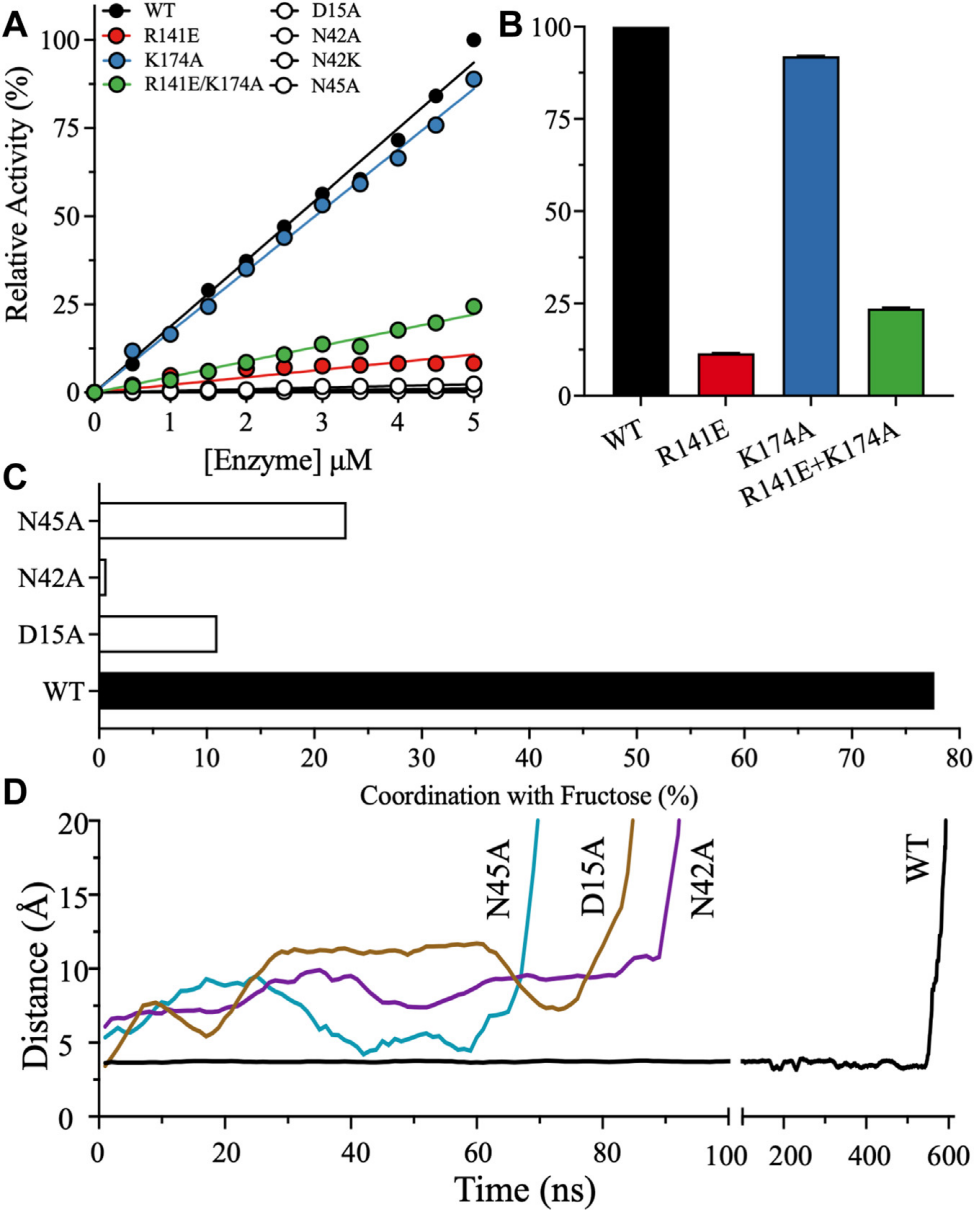
**The effects of active site residues on activity and fructose binding.***A*, the relative activities of WT and mutant KHK-A were measured at different enzyme concentrations and fixed saturating concentrations of substrates (20 mM fructose and 5 mM ATP). *B*, bar plots of relative enzyme activity that are determined from the slopes of enzyme rates. Data are the mean ± SD, n = 3. *C*, comparison of percentage of coordination between D258 and fructose in WT KHK-A *versus* inactive mutants. *D*, the distance between D258 and fructose in the closed state shows that fructose leaves more quickly from the inactive mutants than WT. KHK, ketohexokinase.

To elucidate the contributions of residues in the fructose-binding pocket to substrate binding and the overall catalytic function of KHK-A, alanine substitutions were introduced at residues D15, N42, N45, and K174 (Fig. 4, *A* and *B*). These residues all directly interact with fructose and were expected to contribute to the binding interactions between fructose and the active site. However, these residues do not interact with the C1 hydroxyl of fructose, which is the site of phosphorylation by ATP, and thus we did not expect these mutations to eliminate catalytic activity.

The mutant proteins were expressed and purified as described for WT KHK-A. The activity of WT and mutant KHK-A was monitored using a pyruvate kinase and lactate dehydrogenase (PK/LDH) coupled-enzyme assay under conditions of fructose and ATP saturation. Surprisingly, the incorporation of alanine substitutions at D15, N42, and N45 completely inactivated KHK-A. R141E and K174A retained 12% and 92% of WT activity (Fig. 4, *A* and *B*). The relatively high activity of K174A may be attributable to coordination between E173 and the C6 hydroxyl with which K174 interacts.

The MD simulation indicated that R141 functions as an anchor to seal the catalytic pocket upon substrate entry (Fig. 2, *B* and *C*). RK, another member of the pfkB family, has a glutamate residue (E154) at this position, suggesting that the function of R141 is unique to KHK-A. To mimic the residue at this location in RK, R141 was mutated to glutamate. The R141E mutant and R141E/K174A double mutant had 12% and 24% of the activity of WT KHK-A, respectively (Fig. 4, *A* and *B*).

Similar to the switch from R141 in KHK-A to E154 in RK, N42 of KHK-A corresponds to a lysine in RK (K54). Because KHK-A N42A was completely inactive, a lysine mutation was introduced at N42 to potentially restore activity and gain deeper insights into the catalytic mechanism of KHK-A. Interestingly, the N42K mutation did not restore KHK-A activity.

The structural integrity of the KHK-A mutants was subsequently probed by far-UV CD analysis, which yielded spectra with dual ellipticity minima at 208 nm and 222 nm, identical to WT (Fig. S1*A*). To further evaluate the oligomerization state of the KHK-A variants, MP was performed. MP is a single-molecule technique that can be used to estimate the mass distribution of a protein in its native state by quantifying the light scattering produced by protein particles in solution. All measurements were conducted using 100 nM protein diluted in PBS. The data acquisition time was 60 s for all measurements, which typically yielded ∼3000 binding events. The interferometric contrast measurements obtained by MP were converted to mass measurements by reference to a standard curve constructed using protein standards of known molecular weight: monomeric creatine kinase (46.7 kDa), bovine serum albumin (66 kDa, 132 kDa, and 198 kDa), and alcohol dehydrogenase (150 kDa). The histogram data from the experiment were converted to a probability density curve *via* kernel density estimation (Fig. S1*B*). The mass distribution of WT yielded a large peak at ∼69 kDa, which corresponds to the dimer form of KHK-A, as the theoretical molecular weight of the monomer is 34.65 kDa. All KHK-A mutants exhibited a similar large peak at ∼69 kDa, indicating a dimeric state (Fig. S1*B*). Thus, despite partially or completely disrupting activity, the mutations had minimal effects on the oligomeric state of KHK-A.

### MD simulation on mutants in the fructose-binding pocket of KHK-A

The inactive mutants were further analyzed *in silico* to provide mechanistic insights into the impact of the mutations on ligand coordination and enzyme dynamics. Residues at the fructose-binding site were considered to interact with fructose if the distance between the residues and fructose was less than 6 Å. The corresponding counts during the entire simulation time were converted into a percentage of coordination between the residues and fructose. Compared to WT KHK-A, D12A, N42A, and N45A displayed reduced coordination of the catalytic residue D258 with fructose (Fig. 4*C*). Throughout the simulation run, WT exhibited 78% coordination between D258 and the C1 hydroxyl of fructose, whereas D15A, N42A, and N45A had 11%, 1%, and 23% coordination, respectively. Compared to WT KHK-A, the overall coordination between fructose and active site residues such as D15, N42, N45, K174, and D258 was lower in the inactive mutants tested here (Fig. S3*C*). This large decrease in fructose coordination explains the catalytic inactivation of the D15A, N42A, and N45A mutants.

MD simulations confirmed that fructose left the substrate-binding site much earlier in the inactive mutants than in WT KHK-A (Fig. 4*D*). Furthermore, dynamic cross-correlation map (DCCM) analyses of WT KHK-A revealed that in the open state, the region surrounding ATP was decoupled from the region surrounding the fructose-binding site. This decoupling led to increased positive correlations in the ATP-binding region and increased negative correlations between ATP and the fructose-binding region, as evidenced by brighter cyan and pink coloring, respectively, in the DCCM plots (Fig. S4). By contrast, the region surrounding ATP and the fructose-binding site were coupled in the closed state, resulting in smaller modulations in the correlations and muted coloring in the DCCM plots. Collectively, these data support cooperativity between the two protomers, with one remaining in the open state and the other in the closed, substrate-bound state. In other words, one protomer facilitates the reaction while the other prepares for the next reaction.

### Initial velocity studies of WT and mutant KHK-A

**Figure 5:**
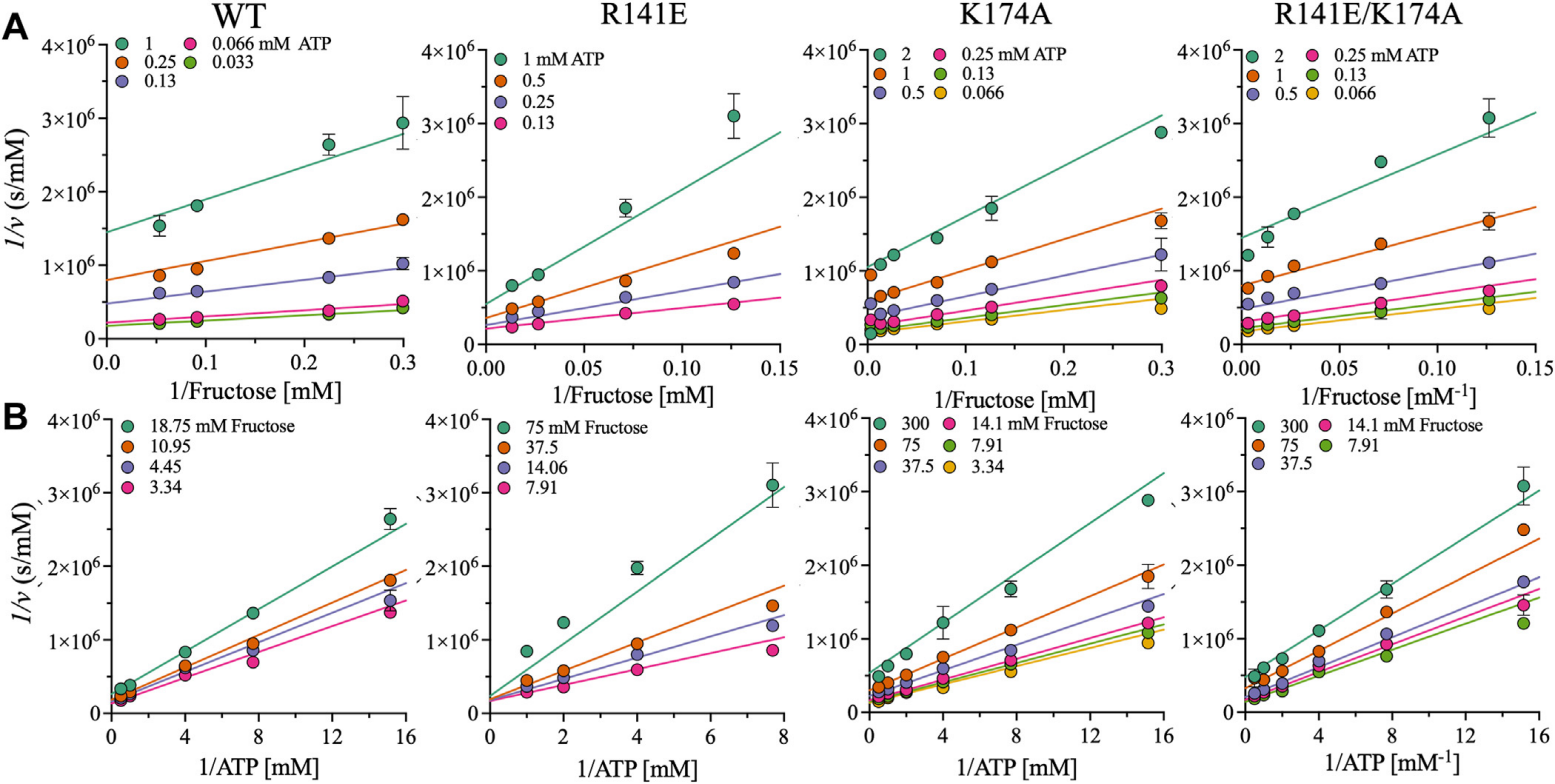
**Initial velocity studies of WT and mutant KHK.***A* and *B*, double-reciprocal plots of the initial velocities of WT and mutant (R141E, K174A, and R141E/K174A) KHK-A. The initial velocities were determined by varying the fructose concentration at different fixed ATP concentrations. The points on the graphs represent experimental data, and the lines are the theoretical fit of a bi-bi sequential mechanism. Data are the mean ± SD, n = 3. KHK, ketohexokinase.

Most of the mutations were located in the fructose-binding pocket and resulted in inactivation. Thus, initial velocity studies were limited to WT KHK-A and the active mutants: R141E, K174A, and R141E/K174A. The fructose concentrations were varied at different fixed ATP concentrations, and the kinetic parameters were extrapolated from the initial velocity measurements in the forward direction. The initial velocity data were fit to a random Bi–Bi sequential kinetic framework (Fig. 5, *A* and *B*). The catalytic rates (*V*/*E*_t_) of R141E, K174A, and R141E/K174A were similar to that of WT (Table S2), as were the Michaelis constants for ATP (*K*_ATP_). However, the Michaelis constants of the mutants for fructose (*K*_Fru_) were 4-fold higher than that of WT, indicating a reduction in fructose affinity with no change in ATP affinity. R141E/K174A had the lowest *V/E*_*t*_ and highest *K*_Fru_. Overall, *V/K*_Fru_*E*_t_ was 3- to 10-fold lower for the mutants than for WT, whereas *V/K*_ATP_*E*_t_ did not change significantly, with a decrease of less than 3-fold (Table S2).

### Thermodynamic stability of KHK-A catalytic pocket mutants

**Figure 6:**
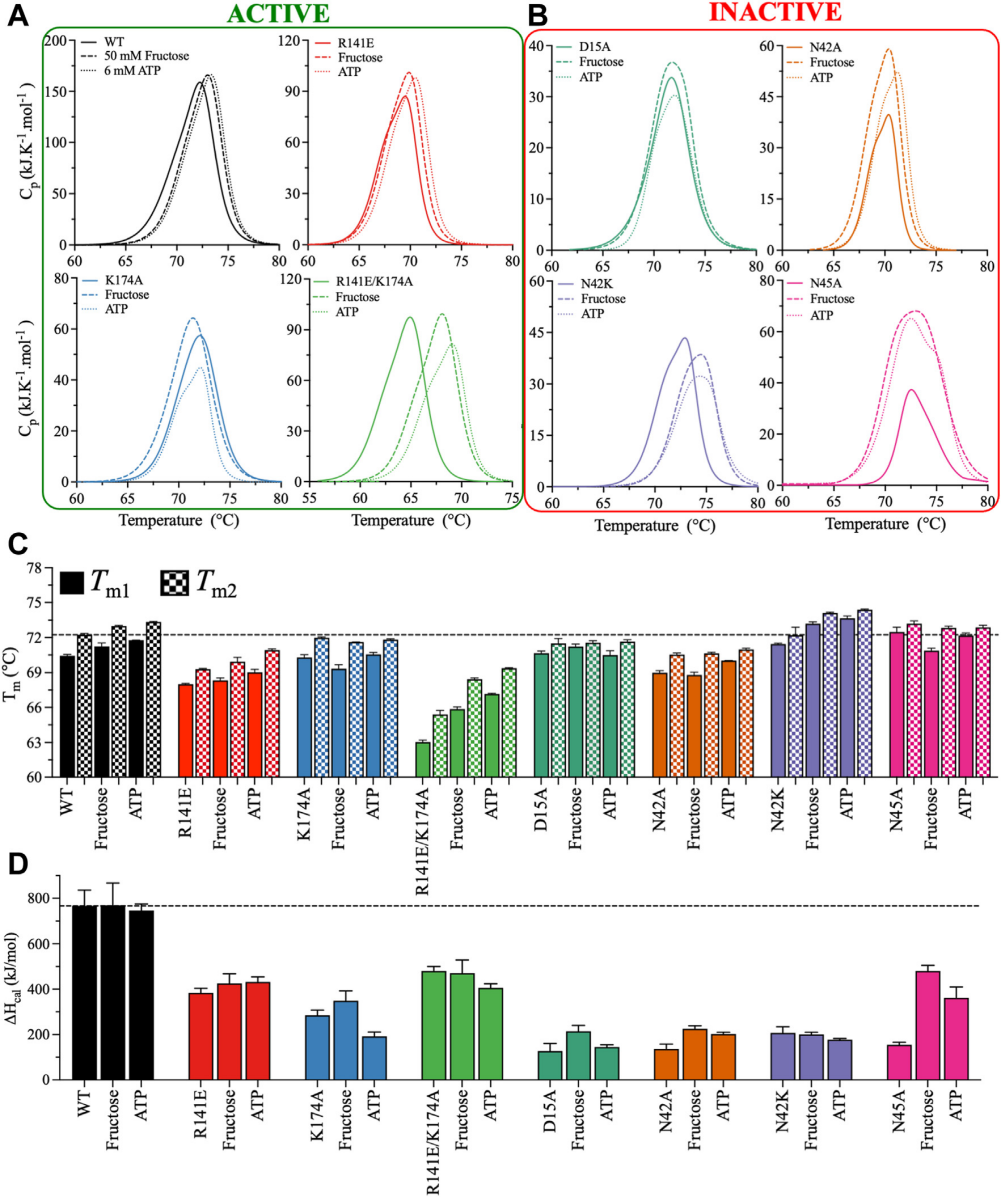
**Thermodynamic stability of WT and mutant KHK-A in the presence and absence of substrate.***A*, DSC thermograms of the active variants in the absence or presence of substrate (50 mM fructose or 6 mM ATP): WT (*black*), R141E (*red*), K174A (*blue*), and R141E/K174A (*green*). *B*, DSC thermograms of the inactive variants in the absence or presence of substrate (50 mM fructose or 6 mM ATP): D15A (*teal*), N42A (*orange*), N42K (*purple*), and N45A (*pink*). The DSC scans were corrected for the buffer baseline, and the data were fit to a single-state transition. The thermograms were deconvoluted using the Nano-Analyze software package from TA instruments. *C* and *D*, bar plots of *T*_m1_, *T*_m2_ and *ΔH*_cal_ in the absence and presence of substrate (50 mM fructose or 6 mM ATP). Data are the mean ± SD, n = 3. DSC, differential scanning calorimetry; KHK, ketohexokinase.

DSC was performed to analyze the thermodynamic stability of the KHK-A mutants (Fig. 6). The calorimetric enthalpy (*ΔH*_cal_) was calculated by integrating the area under the thermal peak, which corresponds to the total heat absorbed by the protein during heat-induced denaturation.

The unfolding transitions of WT and mutant KHK-A were monitored in the absence or presence of 50 mM fructose (10 × *K*_Fru_) or 6 mM ATP (10 × *K*_ATP_). To ensure that high fructose concentrations did not affect thermodynamic stability, DSC thermograms of the WT enzyme were first obtained at fructose concentrations of 1 mM, 10 mM, and 50 mM (Fig. S5*A*). The WT enzyme exhibited similar *T*_m_ and *ΔH*_cal_ values at different fructose concentrations, validating the use of the saturating fructose concentration of 50 mM in the DSC scans of the mutant enzymes. The thermal profiles of all KHK-A mutants were similar to those of WT, with deconvolution patterns revealing the presence of a single-state thermal transition yielding one melting temperature (*T*_m_). Figure 6*A* shows the results for the active mutants (R141E, K174A, and R141E/K174A), while Figure 6*B* shows the results for the inactive mutants (D15A, N42A, N42K, and N45A) (Fig. 6, *A* and *B*). The melting temperature (*T*_m_) of WT did not change in the absence or presence of substrate, with values of 72.2 ± 0.1 °C, 73.0 ± 0.1 °C, and 73.3 ± 0.1 °C in the absence and presence of fructose or ATP, respectively (Fig. 6*C*).

The introduction of the R141E mutation greatly altered the thermal characteristics of the apo state. Compared with WT, the R141E mutation reduced *T*_m_ by approximately 2 to 4 °C in the absence or presence of substrates. The *T*_m_ of R141E shifted from 69.3 ± 0.1 °C for apo-R141E to 70.0 ± 0.3 °C and 70.9 ± 0.1 °C in the presence of fructose or ATP, respectively (Fig. 6*C*). The *T*_m_ of K174A was comparable to that of WT in the apo state but decreased by ∼1.5 °C upon the addition of the substrate. The double mutant R141E/K174A exhibited the greatest reductions in *T*_m_, with values of 65.4 ± 0.3 °C, 68.4 ± 0.1 °C, and 69.4 ± 0.1 °C in the absence or presence of fructose or ATP, respectively. The *T*_m_ values of the inactive mutants D15A, N42A, and N45A were similar and did not change significantly upon substrate binding. However, compared to WT, the N42K mutation increased *T*_m_ by approximately 1 °C in the presence of either substrate (Fig. 6*C*). All mutants exhibited decreases in *ΔH*_cal_ of 2- to 6-fold compared to WT, irrespective of the presence or absence of substrate (Fig. 6*D*). These observations imply that the mutations in the fructose-binding pocket increased the overall hydrophobicity of the protein.

We also investigated the relationship between temperature and relative activity. The DSC analysis indicated that WT and mutant KHK-A have high thermodynamic stability, suggesting that elevated temperatures would be needed to inactivate the enzyme. However, the PK/LDH coupled-enzyme assay used to monitor KHK-A activity is incompatible with high temperatures, as the *T*_m_ of PK is relatively low, 48 °C (45). Consequently, we studied the effect of preincubation temperature on the activity of WT and mutant KHK-A. We defined the half-inactivation temperature (IT_1/2_) as the incubation temperature at which enzyme activity was reduced by half. To determine IT_1/2_, each KHK-A variant was incubated for 10 min at different fixed temperatures ranging from 40 °C to 80 °C, and activity was then measured at 37 °C in the presence of the coupled enzymes, PK and LDH, and fixed saturating concentrations of fructose and ATP. The enzymatic activity of each sample was normalized to its activity at 25 °C. The same enzyme concentration was used for WT KHK-A and the mutants.

**Figure 7:**
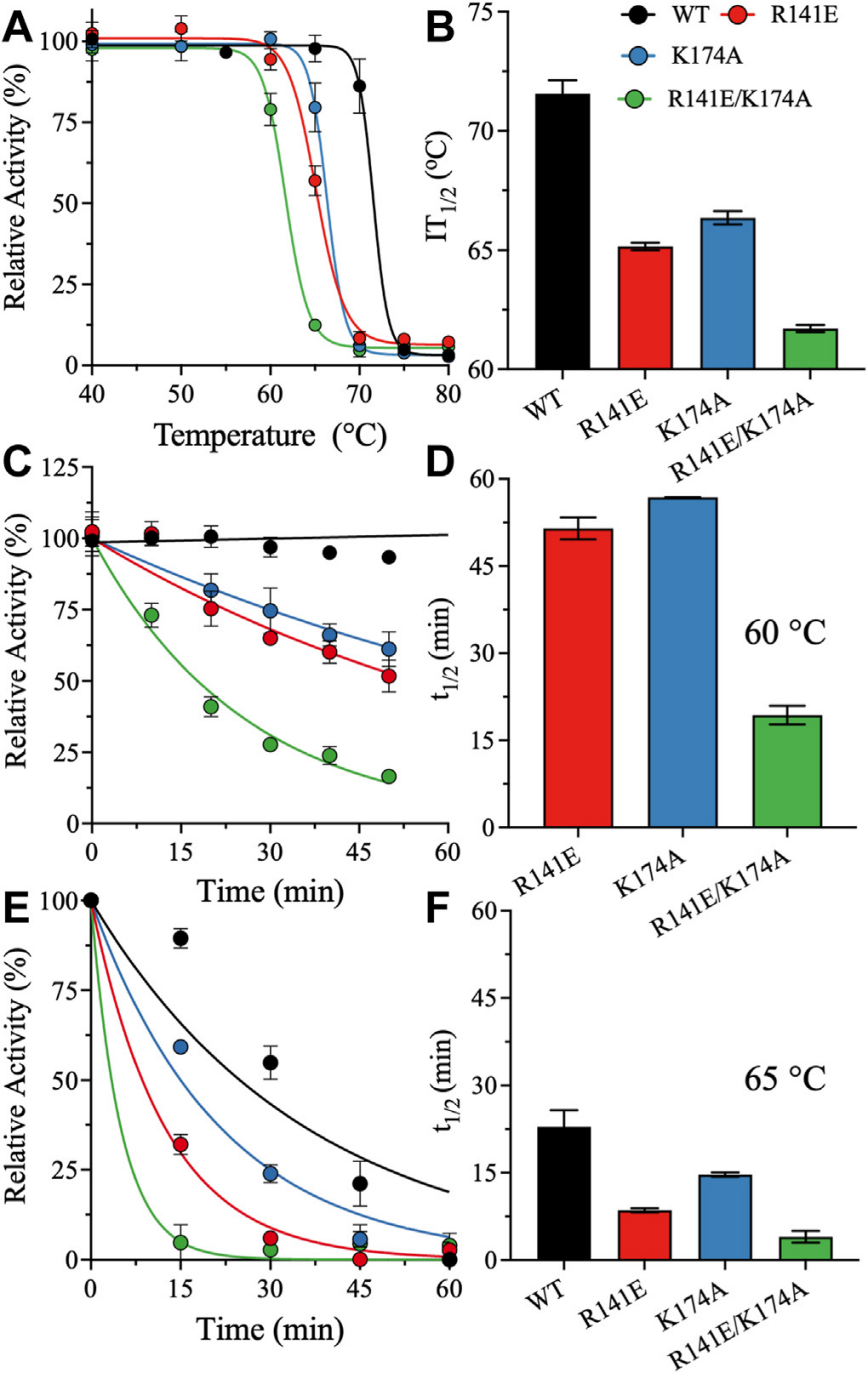
**Half-inactivation temperature (IT**_**1/2**_**) and half-life (t**_**1/2**_**) of WT and mutant KHK-A.***A*, the kinetic stability of WT and mutant KHK-A as a function of incubation temperature. Enzymatic activity was measured after incubating KHK-A at different temperatures for 5 min. Relative activity was normalized against the catalytic rate when the enzyme was incubated at 37 °C. *B*, bar plots of IT_1/2_ that were determined from the half-transition of the graphs in panel “A”. *C* and *E*, the residual enzymatic activity of KHK-A at different time intervals after incubation at 60 °C and 65 °C. *D* and *F*, bar plots of *t*_1/2_ of KHK-A when incubated at 60 °C and 65 °C. Data are the mean ± SD, n = 3. IT_1/2_, half-inactivation temperature; KHK, ketohexokinase.

WT exhibited robust stability, with unaltered activity after incubation at temperatures of up to 68 °C; at higher incubation temperatures, activity gradually declined, and IT_1/2_ was 72 °C (Fig. 7, *A* and *B*). By contrast, R141E and K174A maintained activity up to incubation temperatures of 60 °C, with IT_1/2_ values of 65 °C and 67 °C, respectively (Fig. 7, *A* and *B*). Notably, the double mutant, R141E/K174A, retained 100% activity only up to 55 °C, with IT_1/2_ at 62 °C (Fig. 7, *A* and *B*). Consistent with the DSC scans, these observations imply that the mutants were less stable than WT KHK-A.

To further assess the effects of the mutations on the structural stability of KHK-A, the *t*_1/2_ values for inactivation of the WT and mutant enzymes at fixed incubation temperatures of 60 °C and 65 °C were determined. WT and mutant KHK-A were incubated at fixed temperatures for time intervals of up to 60 min, and enzymatic activity was measured using the PK/LDH coupled-enzyme assay at fixed saturating concentrations of fructose and ATP. Incubation at 60 °C for 60 min did not alter the catalytic activity of WT KHK-A; thus, *t*_1/2_ could not be calculated. R141E and K174A were fairly stable, with *t*_1/2_ values of 51.5 ± 1.9 min and 56.8 ± 0.1 min, respectively (Fig. 7, *C* and *D*). By contrast, the *t*_1/2_ of R141E/K174A was far lower: 19.4 ± 1.6 min. At 65 °C, *t*_1/2_ was 22.9 ± 2.3 min for WT KHK-A, 8.5 ± 0.3 min for R141E, 14.7 ± 0.3 min for K174A, and 2.9 ± 0.6 min for R141E/K174A (Fig. 7, *E* and *F*). Overall, the WT enzyme had higher thermal stability and *t*_1/2_ values than the mutants at both incubation temperatures. Collectively, these observations and the DSC results underscore the importance of R141 and K174 for the thermodynamic stability of KHK-A.

### Protein–protein interaction network associated with KHK-A and its involvement in tumors

The global protein–protein interaction (PPI) network represents the physical contacts and associations between proteins, crucial for cellular functions. PPI networks provide insights into cellular organization and dynamics, aiding in understanding KHK-A's role in disease mechanisms. The KHK-A PPI network includes 448 proteins with 9711 connections in three modules. A subnetwork of 23 proteins, connected by 79 interactions, links to three clusters. Except for fructokinase (FCSK), these proteins show differential expression (*p*-value < 0.01) between tumors and healthy tissue (GENT2 database) (46).

**Figure 8:**
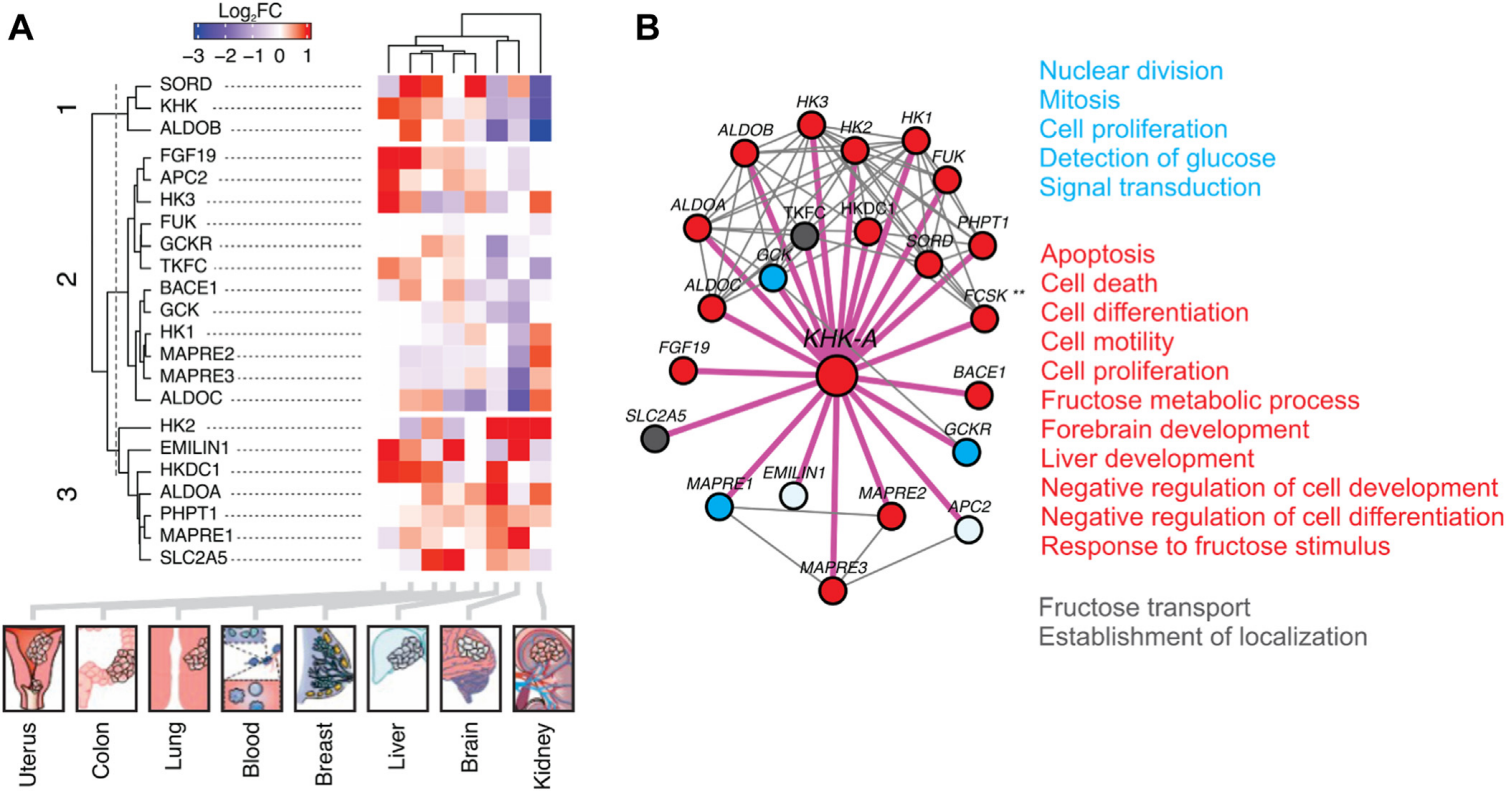
**PPI network of KHK-A and proteins involved in different types of cancers.***A*, the heatmap displays the fold change (FC) in the expression of genes that are differentially expressed between tumor and healthy tissues (*p* value < 0.01) in various organs (lung, uterus, blood, colon, breast, prostate, liver, brain, and kidney). The encoded proteins directly interact (first degree) with KHK-A. Different shades of *blue* in the heatmap indicate reduced expression in tumor tissues compared to healthy tissues, while shades of *red* indicate higher expression in tumor tissues. *B*, the PPI network highlights first-degree interactions with KHK-A with *thick lines* (*pink* connectors) and second-degree interactions with *thin lines* (*black* connectors). Of all the proteins that interact directly with KHK-A, only FCSK (∗∗) did not show differential expression associated with any cancers in which KHK has significant differences. KHK, ketohexokinase; PPI, protein–protein interaction.

Heatmap analysis reveals KHK downregulation in the kidney (FC = −2.43), the liver (FC = −1.05), the brain (FC = −0.85), and blood (FC = −0.22), but upregulation in the breast (FC = 0.18), the colon (FC = 0.68), the lung (FC = 0.36), and the uterus (FC = 0.86) tumors. Proteins interacting with KHK, such as ALDOC, MAPRE2, GCK, HK1, and BACE1, showed varied expression, with consistent downregulation in the brain. Notably, GCK, involved in glucose catabolism and immune responses, interacts with KHK (Fig. 8, *A* and *B*).

A third group, including HK2, EMILIN1, SLC2A5, HKDC1, ALDOA, PHPT1, and MAPRE1, also shows differential expression. For example, HK2 is downregulated in blood and colon but upregulated in the kidney, brain, liver, and lung. EMILIN1 is upregulated in the uterus, the brain, blood, and the colon, but downregulated in the lung, kidney, and liver. SLC2A5 is downregulated in the kidney and colon, with similar trends observed for other proteins in this cluster.

## Discussion

The consumption of foods with high fructose content is strongly associated with obesity and MetS and thus poses a serious threat to global health (6, 7, 8, 10, 11, 12, 16, 17, 18, 19). MetS increases the risk of developing type 2 diabetes and insulin resistance because cells become less responsive to the effects of insulin (47). Unlike glucose, fructose does not stimulate a robust insulin response. Fructose is transported to the liver *via* GLUT2 and phosphorylated by KHK in the presence of ATP to yield F-1P (48), which is subsequently converted to triglycerides and stored as fat; thus, high fructose consumption is also linked to nonalcoholic fatty liver disease (NAFLD) (49). Understanding the molecular transport and metabolism of fructose is essential for developing therapies for MetS and NAFLD.

Fructolysis is far less tightly regulated than glycolysis. Glucose metabolism is regulated by two key enzymes in the first stage of glycolysis: hexokinase and PFK (50, 51). Hexokinase is feedback-inhibited by its product, glucose-6-phosphate. PFK, which catalyzes the rate-limiting step of glycolysis and is negatively regulated by the presence of ATP, contributes to cellular energy homeostasis and maintaining adequate cellular ATP levels. Unlike hexokinase, KHK is not controlled by feedback inhibition or allosteric regulation and is not rate-limited (24). F-1P, the product of KHK, bypasses PFK and is cleaved by aldolase B into glyceraldehyde and dihydroxyacetone phosphate, which are further metabolized in the second stage of the glycolytic pathway (52). As a result, fructolysis bypasses two key enzymatic steps that are important in regulating glycolysis. Elevated KHK activity in the liver due to fructose consumption may lead to hepatic ATP depletion, disrupting cellular functions, and metabolic processes (53, 54). These effects can potentially contribute to liver injury and inflammation.

The general structural fold of kinases in the pfkB family consists of a large central α/β domain and a protruding lid domain comprising a four-stranded β-sheet (36). KHK-A is an elongated homodimer with its lid domain facilitating dimerization through the formation of the β-clasp structure (34, 37). The β-clasp resembles a “handshake” and is found in other (but not all) members of the pfkB family, including RK, adenosine kinase, tagatose-6-phosphate kinase, and the other human KHK isozyme, KHK-C (34, 36, 37, 38, 55). The β-clasp is not only responsible for dimerization but also acts as a lid on the fructose-binding pocket. Comparing our apo-KHK-A crystal structure with KHK-A (PDB code: 2HW1) in complex with fructose and adenylyl imidodiphosphate (AMP-PNP), an ATP analog, demonstrated no significant differences in the overall structural fold of KHK-A upon substrate binding (37). Minor conformational differences have been observed at the loop (Y113-S116) after β7, the last β-strand of the β-clasp (37). However, it is documented that the crystal structures of *E. coli* RK displayed open- and closed-states in the absence and presence of substrates (56). Our MD simulation also displays the same phenomenon. It revealed a cooperative mechanism of the β-clasps that involves opening one protomer while closing the other. This ensures substrate processing at one catalytic site at a time in the KHK-A dimer.

Of the five residues in the fructose-binding pocket of KHK-A that were mutated in this study, only D15 is part of the β-clasp; the others are part of the large central α/β domain. The introduction of alanine substitutions at three of the five residues—D15, N42, and N45—completely inactivated KHK-A. D258 is the catalytic residue, and D15, N42, and N45 were expected to have greater effects on binding than on catalysis. The apparently essential roles of these residues in the catalytic mechanism of KHK-A can be explained by the significant reduction in fructose coordination when these residues were mutated, as observed in the MD simulations. Fructose left at much earlier times in D15A, N42A, and N45A than in WT, further supporting the notion that mutations of these residues significantly reduce fructose affinity.

We observed a high *K*_Fru_ for the WT KHK-A, consistent with a previously reported value and well above serum fructose concentrations (31, 57). KHK-C has a far lower *K*_Fru_ value than KHK-A (31). Because KHK-A is a homodimer, it is impossible to experimentally examine the contribution of each protomer to enzymatic activity; thus, the occupancy state of KHK-A remains unclear. Atomistic MD simulations with doubly occupied monomers demonstrated that fructose left one protomer shortly after the simulation started, while the other protomer remained occupied throughout the 600-ns simulation time. A detailed investigation of the trajectories showed that the empty protomer approached the other from its β-clasp, thus enabling the occupied protomer to adopt a more compact binding pocket (Video S1). Importantly, in the crystal structure of KHK-A in complex with fructose and AMP-PNP (PDB code: 3NBV), only one of the protomers is occupied by fructose (58).

The catalytically active mutants in the fructose-binding pocket were characterized to investigate further their roles in fructose binding and catalysis of KHK-A. Introducing alanine at R141 and K174 only partially inactivated KHK-A. The *K*_Fru_ of K174A was 2.4-fold higher than that of WT. K174 may form weak hydrogen bonding interactions at 4.9 Å with the C6 hydroxyl of fructose. The elevated *K*_Fru_ of K174A indicates that K174 is important for establishing bonding interactions with fructose that facilitate its binding. Additionally, *V*/*E*_t_ was lower for K174A than for WT KHK-A, suggesting that K174 functions in both fructose binding and catalysis. *K*_ATP_ did not differ significantly between WT KHK-A and the catalytically active mutants, consistent with the lack of proximal interactions between the mutated residues and ATP in the crystal structure.

To determine if the mutations of fructose-binding pocket residues affected substrate binding, DSC was performed in the presence and absence of substrates. The partially active mutant K174A exhibited a significant decrease in *ΔH*_*cal*_ compared to WT, and even larger decreases in *ΔH*_*cal*_ were observed for the inactive variants. Decreases in *ΔH*_*cal*_ are often attributed to increased exposure of a protein’s hydrophobic core, as hydrophobic interactions contribute exothermically to *ΔH*_*cal*_ in polar solutions (59). The inverse correlation between *ΔH*_*cal*_ and protein hydrophobicity is well documented (60, 61, 62, 63). The decreases in *ΔH*_*cal*_ for D15A, N42A, N42K, and N45A suggest large changes in protein hydrophobicity, which may explain their inactivity.

KHK-A is more thermodynamically stable than KHK-C (31). Our analysis of thermal inactivation kinetics revealed that incubating KHK-A for 30 min at 60 °C reduced its activity by only 10%, whereas incubating KHK-C for 30 min at 40 °C reduced its activity by 50% in a previous study (31). In this study, the activity of KHK-A decreased by 50% only at ∼70 °C. Additionally, despite having similar *T*_m_ values as WT KHK-A, K174A, R141E, and R141E/K174A had lower *t*_1/2_ values and reached 50% activity at lower incubation temperatures. This instability at high temperatures may be explained by the higher hydrophobicity observed by DSC.

Residues D15, N42, N45, R141, and K174 are conserved between KHK-A and KHK-C; therefore, targeting these residues would certainly affect the catalytic activity of both isozymes. These residues are attractive targets for drug discovery efforts. In animal models, knocking out KHK mitigates metabolic defects, and KHK KO mice remain healthy and show no biochemical abnormalities, suggesting that reducing KHK activity is an alternative to reducing fructose consumption. Additionally, knocking out KHK in mice protects against hepatic steatosis and improves insulin sensitivity (64). Other studies have shown that high-fructose diets upregulate KHK, promoting lipogenesis (65). Pharmacological inhibition of KHK also hampers the development of type 2 diabetes and NAFLD (66).

In addition to acting as a sugar kinase, KHK-A functions as a protein kinase to promote cancer proliferation. KHK-A is involved in the phosphorylation and activation of phosphoribosyl pyrophosphate synthetase 1 and 14-3-3 eta protein, which promotes nucleic acid synthesis in hepatocellular carcinoma and triggers cell migration in breast cancer (1, 67). As a protein kinase, KHK-A probably plays important roles in other signaling pathways. Overall, studies suggest that KHK-C is critically involved in cardiometabolic complications, whereas KHK-A is a key player in tumor progression (1, 65, 67, 68). Thus, our analysis of the crystal structure of human KHK-A and its fructose-binding pocket may also have implications for cancer treatment.

The interactome, which represents PPI and the gene expression in the heatmap analyses of KHK, provided critical insights into its role in cancer development. KHK expression is downregulated in tumors in the blood, liver, brain, and kidney. Some of the KHK interacting partner proteins also showed changes in their expression, including HK2, EMILIN1, SLC2A5, HKDC1, ALDOA, PHPT1, and MAPRE1. Notably, HK-2 and EMILIN1 are upregulated in brain tumors. While the gene expression of HK2 is upregulated and EMILIN1 is downregulated in tumors in the kidney, liver, and lungs. These organ-specific expression patterns call for a reexamination of KHK's role in blood, lung, liver, brain, uterus, and kidney cancers to determine whether KHK-A is a potential target for preventing cancer metastasis. Previous studies have indicated the roles of KHK-A’s interacting partner proteins in cancer (69, 70). For example, EMILIN1 suppresses tumor growth, and deregulation of EMILIN2 in gastric cancer promotes tumor growth and angiogenesis (71). The fructose transporter SLC2A5 is upregulated in the lung and blood while downregulated in the kidney and colon. Also, the loss of SLC2A5 has been shown to inhibit cancer cell migration (72). The genes GCK, MAPRE3, and MAPRE2 hold promise for guiding therapeutic strategies for organ-specific cancers. To develop robust strategies for cancer diagnostics and treatment, it is imperative to visualize PPIs in an organ-specific manner.

Overall, high glucose consumption promotes the role of KHK in fatty liver disease, metabolic disorders, and cancer, where inhibiting KHK effectively hampers the growth and movement of glioma cells in the presence of fructose (73). Therefore, novel therapeutic approaches focusing on KHK inhibition and addressing fructose intake are potential strategies for treating glioma. By doing so, this study highlights the potential of the KHK-A signaling pathway and its partner proteins as cancer targets.

## Experimental procedures

### Protein expression and purification

Sequences encoding WT or mutant KHK-A with an N-terminal Hisx6 tag were introduced into the pET-28b (+) bacterial expression vector by GenScript, Inc. The resulting vectors were used to transform *E. coli* BL21-CodonPlus-RIL (Stratagene). For protein expression, 100 ml of overnight culture grown in LB medium was used to inoculate 1.8 L of terrific broth medium supplemented with 50 μg/ml kanamycin and chloramphenicol. The culture was incubated with shaking at 37 °C, and when the *A*_600_ reached ∼3.0, protein expression was induced by adding 1 mM IPTG. The cultivation temperature was then reduced to 15 °C, and the cells were allowed to grow overnight before harvesting and flash freezing. The cell pellet was suspended in 100 ml of binding buffer (10 mM Tris pH 7.5, 150 mM NaCl, 5 mM imidazole, and 3 mM β-mercaptoethanol) supplemented with a protease inhibitor cocktail (0.1 mM benzamidine-HCl and 0.1 mM phenylmethyl sulfonyl fluoride). The lysate was centrifuged at 15,000 rpm for 30 min, and the supernatant was loaded onto a 3-mL Ni-NTA column (Qiagen) equilibrated with binding buffer at 4 °C. The Ni-NTA column was washed with 150 ml of wash buffer (10 mM Tris pH 7.5, 150 mM NaCl, 30 mM imidazole, and 3 mM β-mercaptoethanol) and eluted with wash buffer supplemented with 300 mM imidazole. The protein was further purified by gel filtration on a Superdex 200 pg (16/600) column preequilibrated with 20 mM Hepes pH 7.5, 150 mM NaCl, and 0.5 mM tris(2-carboxyethyl)-phosphine. An Amicon Ultra centrifugal filter was used to reduce the final protein concentration to 3 mg/ml for biochemical assays and 37 mg/ml for crystallization trials. Protein concentrations were measured using the Bradford assay, and protein purity was > 95% as assessed by SDS-PAGE.

### Determination of oligomeric state by CD and mass photometry

The structural fold and secondary structure of KHK-A were characterized by CD spectroscopy. CD scans of WT and mutant KHK-A were collected in 100 mM phosphate buffer (pH 7.5) at a scanning rate of 1 nm/s and wavelength range of 200 to 260 nm. The protein concentration was 0.4 mg/ml, and the samples were scanned in 1-mm quartz cuvettes at 25 °C in a Chirascan CD spectrometer (Applied Photophysics). The spectrometer was calibrated with aqueous camphor-10-sulfonic acid.

MP measurements were performed in a TwoMP mass photometer (Refeyn Ltd) at room temperature. Microscope coverslips (Refeyn Ltd) were cleaned with isopropanol and Milli-Q water, and a reusable six-well silicone gasket was mounted at the center of each coverslip. An 18-μL drop of PBS was added to each well to allow focusing of the lens on the glass surface. For MP mass measurements, contrast mass calibration was performed using different proteins of known molecular weight in PBS buffer (pH 7.4): creatine kinase (47 kDa), bovine serum albumin (66 kDa, 132 kDa, and 198 kDa), and alcohol dehydrogenase (150 kDa). Five data points were used for the mass calibration. The distribution of light-scattering events, expressed as interferometric contrast, was fit to a Gaussian distribution using DiscoverMP software (Refeyn Ltd, https://www.refeyn.com/software-release-updates). The contrast values were converted to masses by establishing a linear relationship between the contrast and mass of the binding object from the standard set of proteins. The accepted R2 values of the standard curves were greater than 0.999. The WT and mutant KHK-A samples were diluted to a concentration of 100 nM in PBS (74). The acquisition time for data collection was fixed at 60 s. Data were collected using AcquireMP software (Refeyn Ltd) and were further processed using DiscoverMP (Refeyn Ltd), which yielded a distribution histogram of the number of counts of each contrast value (or mass after calibration). The number of binding events in each run was always greater than 3000. Kernel density estimation was applied to the counts to convert the histogram into a curve.

### Crystallization and structural determination

Crystals of WT KHK-A were grown by sitting-drop vapor diffusion in a mixture of 19% PEG1500, 0.2 M NH_4_SO_4_, 5 mM CoCl_2_, and 0.1 M sodium citrate, pH 4.2, at 18 °C. The crystals reached a size of approximately 100 μm within one to two days. Diffraction data were collected on a Rigaku FR-E with a Rigaku RAXIS image plate at a wavelength of 1.5418 Å and processed using the HKL2000 suite (75). The crystal structure was solved using Phaser (https://www.ccp4.ac.uk/html/phaser.html) in the CCP4 program suite and the molecular replacement method. The structure was refined using REFMAC5. The treatment for the B-factors was isotropic, and the dimer was in the asymmetric unit. The model was deposited with PDB code 2HLZ. Structural figures were prepared using the PyMOL Molecular Graphics System version 1.8 (Schrödinger LLC, https://pymol.org/).

### MD simulation of KHK-A

To examine the structural and dynamic properties of WT KHK-A, D15A, N42A, and N45A, the homodimer form of each variant was first modeled using the solved crystal structure of human apo-KHK-A (PDB: 2HLZ), with each protomer occupied by fructose, ATP, and Mg^2+^. Examination of the crystal structure of human KHK-A, in which fructose was bound in only one protomer, revealed differences between the fructose-occupied protomer and the apo-protomer, especially in the side chains of the residues in the binding site. Despite these differences, our apo-KHK-A was structurally similar to substrate-bound KHK-A (PDB: 3NBV), especially at the binding site. Therefore, fructose, ATP, and Mg^2+^ were introduced into the crystal by referencing the crystal structure of human KHK-A in complex with AMP-PNP and fructose (PDB ID: 3NBV). To model the mutant proteins, we used Schrödinger Maestro software (Schrödinger, Inc, New York, USA, https://www.schrodinger.com/platform/products/maestro/). The WT and mutant model systems were protonated at pH 7.0 using the ProteinPrep tool of the Schrödinger Maestro software. The systems were solvated and neutralized using Solution Builder on the CHARMM-GUI server (76). Charmm36 m (77) and TIP3P (78) were used to model protein and water molecules, respectively, and 0.15 M KCl (76) was used to neutralize the systems.

The WT and mutant systems were simulated using the GROningen Machine for Chemical Simulations (GROMACS) package (https://www.gromacs.org/) (79). The steepest descent algorithm was used to minimize the system before starting the simulations. Next, the temperature was set to 303.15 K using the Nose–Hoover thermostat (80) to perform equilibration in the NVT ensemble for 125 ps. The Nose–Hoover thermostat and Parrinello–Rahman barostat (81) were used to perform MD simulations in the NPT ensemble. The LINCS algorithm (82) was used to constrain H-bonds during minimization, equilibration, and production. Three replicates were performed for each system and initial velocity distribution. Several pair distances between Cα atoms were calculated to show the opening–closing mechanism of the enzyme. Binding site residues were considered to interact with fructose if the distance between the sugar and residues was smaller than 6 Å. The corresponding counts were converted into a bar plot showing the percentage of coordination between the residues and sugar throughout the simulations.

### Dynamic cross-correlation map

Dynamic cross-correlations between Cα atoms were calculated using the Bio3D package (http://thegrantlab.org/bio3d/) (83). The altered correlation motions between the two enzyme protomers were examined using Equation 1:

(1) $$DCC\left(i,j\right)=\frac{<\Delta r_{i}\left(t\right)\cdot \Delta r_{j}\;\left(t\right)\;{>}_{t}}{\sqrt{<{\left|\left|\Delta r_{i}\left(t\right)\right|\right|}^{2}{>}_{t}}\sqrt{<{\left|\left|\Delta r_{j}\left(t\right)\right|\right|}^{2}{>}_{t}}}$$

where the coordinates of the atoms i and j at time t are represented by $r_{i}\left(t\right)$ and $r_{j}\left(t\right)$, respectively. The brackets <> represent the time ensemble average explaining

$$\Delta r_{i}\left(t\right)=r_{i}\left(t\right)-{\left(<\;r_{i}\left(t\right)>\right)}_{t}\text{and}\;\Delta r_{j}\left(t\right)=r_{j}\left(t\right)-{\left(<\;r_{j}\left(t\right)>\right)}_{t}.$$

### Initial velocity and thermal inactivation studies

The KHK-A reaction rate was measured spectroscopically using a PK/LDH coupled reaction. NADH consumption was monitored at 340 nm (ε = 6220 M^−1^⋅cm^−1^) in a 96-well microplate at 37 °C for 10 min in a Cytation 5 multimode microplate reader (BioTek Instruments). The enzymatic reaction contained 20 mM Hepes, pH 7.5, 2 mM MgCl_2_, 0.2 mM NADH, 0.3 mM phosphoenolpyruvic acid, 0.4 U/μl PK, 1.2 U/μl LDH, and different concentrations of fructose and ATP ranging from 0.5 to 10 times their *K*_m_ values. All data were plotted in double-reciprocal form to assess data quality, and the data were fit using Equation (2):

(2) $$v=\frac{V_{\max}AB}{K_{a}K_{b}+K_{b}A+K_{a}B+AB}$$

where *v* and *V*_*max*_ are the initial and maximum velocities, respectively; *A* and *B* are the substrate concentrations; and *K*_*a*_ and *K*_*b*_ are the Michaelis constants for substrates A and B, respectively. The data were fit by global fitting analysis in the kinetics module of SigmaPlot (Systat Software, Inc San Jose, California, www.sigmaplot.com).

To determine the half-inactivation temperature (IT_1/2_), WT or mutant KHK-A was incubated at temperatures between 40 °C and 80 °C for 5 min. Next, 500 nM enzyme was combined with 20 mM Hepes, pH 7.5, 2 mM MgCl_2_, 0.2 mM NADH, 0.3 mM phosphoenolpyruvic acid, 0.4 U/μl PK, and 1.2 U/μl LDH, and KHK-A activity was monitored for 10 min at 340 nm. Error bars were calculated from triplicates of each reaction. IT_1/2_ was determined from the activity remaining after incubation at each temperature. Only active variants were evaluated because inactive mutants cannot be investigated using this approach.

The thermal inactivation kinetics of WT and mutant KHK-A at 60 °C and 65 °C were analyzed using a two-state mechanism (N↔U). Enzyme aliquots were assayed at different time intervals as described above for the determination of IT_1/2_ and plotted as a function of the incubation time, assuming first-order activity decay. Enzymatic activity decay was fit to a first-order reaction (Equation 3) using Prism 10 (GraphPad Software, https://www.graphpad.com/features):

(3) $$\ln\left[A\right]=-kt+\ln\;{\left[A\right]}_{0}$$

where *A*_*0*_ is the initial enzyme activity, *A* is the enzyme activity remaining after incubation time *t*, and *k* is the rate constant for enzyme inactivation. The half-life (*t*_1/2_) is the time required to decrease enzyme activity by half and is equivalent to ln(2)/k.

### Thermodynamic analysis using DSC

Calorimetric thermal unfolding measurements of WT and mutant KHK-A were acquired on a Nano-DSC (TA Instruments) at a protein concentration of 0.6 mg/ml in buffer solution containing 20 mM Hepes, pH 8.0, 150 mM NaCl, and 0.5 mM tris(2-carboxyethyl)-phosphine. All samples were heated from 20 °C to 100 °C at a scanning rate of 1 °C/min under 5 atm pressure in the presence or absence of a single substrate (5 mM ATP or 50 mM fructose). Background scans were collected in buffer (with or without substrates) using the same pressure and temperature parameters described above. The DSC thermograms were corrected by subtracting the corresponding buffer baseline and were converted into plots of excess heat capacity (*C*_p_) as a function of temperature. *T*_m_ was determined at the apex of the thermogram peak, and *ΔH*_cal_ was estimated from the area under the thermal transition, which was fit using the Nano Analyze software (https://www.tainstruments.com/support/software-downloads-support/downloads/) package from TA Instruments.

### System biology and gene expression analysis

A PPI study of KHK-A was conducted and integrated with differential gene expression data for various types of cancer in humans. For interactome studies, a global PPI network for KHK-A was obtained using the STRING databases (84, 85, 86) with a confidence score of 700. The resulting interactome network was analyzed using the Cytoscape program (87) to identify modules using the Molecular Complex Detection plugin (88). Additionally, functional enrichment analysis for each module was conducted using the BiNGO plugin (89) and Cytoscape. A first-degree interaction network of KHK-A was isolated from the global interactome.

The Selenium library programmed in Python was used to obtain differentially expressed genes encoding proteins in the first-degree interaction network of KHK-A from the Gene Expression database of Normal and Tumor tissues 2 (GENT2), available at: http://gent2.appex.kr/. This database contains over 68,000 samples (control and tumor) and provides microarray gene expression data for 72 different tissues. Microarray data for the GPL570 Platform were downloaded and filtered to select genes that were differentially expressed (*p* < 0.01) in conjunction with KHK. Only the tissues in which the KHK-A gene was differentially expressed were selected in this study, and the data were integrated with the PPI data of KHK-A.

## Data availability

The authors declare that all data that support the findings of this study are available within the paper files.

## Supporting information

This article contains supporting information.

## Conflict of interest

The authors declare that they have no conflicts of interest with the contents of this article.
