# Supplementary material for: Residues in the fructose-binding pocket are required for ketohexokinase-A activity
Source: J Biol Chem. 2024 Jul 4;300(8):107538. doi: 10.1016/j.jbc.2024.107538 (PMC11332825; doi:10.1016/j.jbc.2024.107538)
Supplement: Supporting Information [file mmc1.docx]

**Supporting Information**

**Residues in the Fructose Binding Pocket Are Required for Ketohexokinase-A Activity**

Juliana C. Ferreira^1^, Adrian J. Villanueva^1^, Samar Fadl^1^, Kenana Al Adem^1,2^, Thyago H. S. Cardoso^3^, Mario Edson Andrade^4^, Nitin K. Saksena^5^ and Wael M. Rabeh^1, *^

^1^Science Division, New York University Abu Dhabi, PO Box 129188, Abu Dhabi, United Arab Emirates.

^2^Institute of Biological and Medical Imaging, Helmholtz Center München, Helmholtz Association of German Research Centres, Germany

^3^G42 Healhcare Omics Excelence centrer, Masdar City, Abu Dhabi, United Arabes Emirates

^4^Deparmento de Biologia, Universidade Federal de Viçosa, cep 36570-900, Viçosa, Brazil.

^5^Victoria University, Footscray Park Campus, Melbourne, VIC, 3134, Australia.

^*^Corresponding author: wael.rabeh@nyu.edu

Keywords: Ketohexokinase, Fructokinase, Metabolic Syndrome, fructose, obesity, thermodynamic stability, molecular dynamics, kinetics


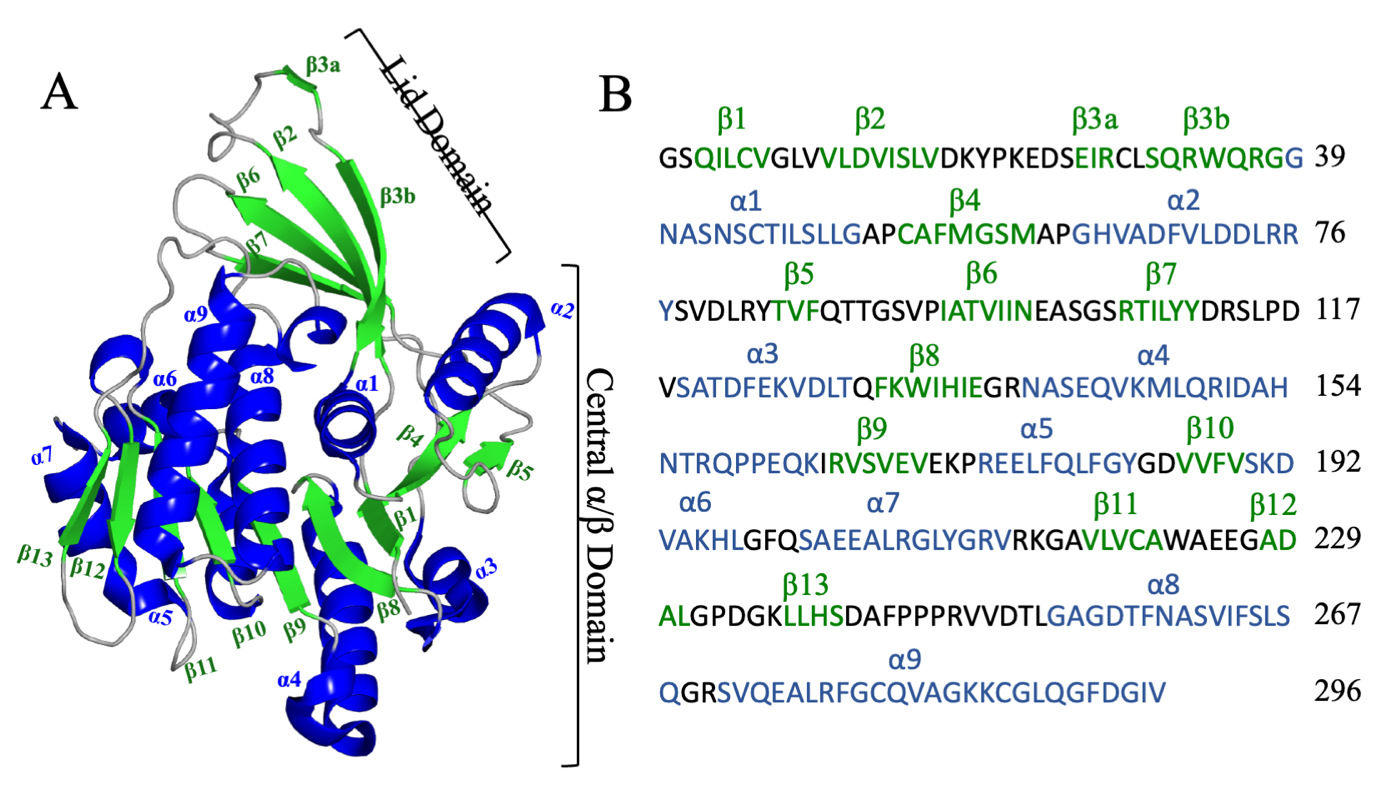


**Figure S1.** **Crystal structure of one monomer of human KHK-A (PDB: 2HLZ).** (**A**) Cartoon representation of the monomeric state of KHK-A that consists of two domains: the central α/β domain and a lid domain. (**B**) Amino acid sequence of KHK-A showing the secondary structural elements that are color coded α-helices (blue) and β-sheets (green). The figure was produced using PyMOL.


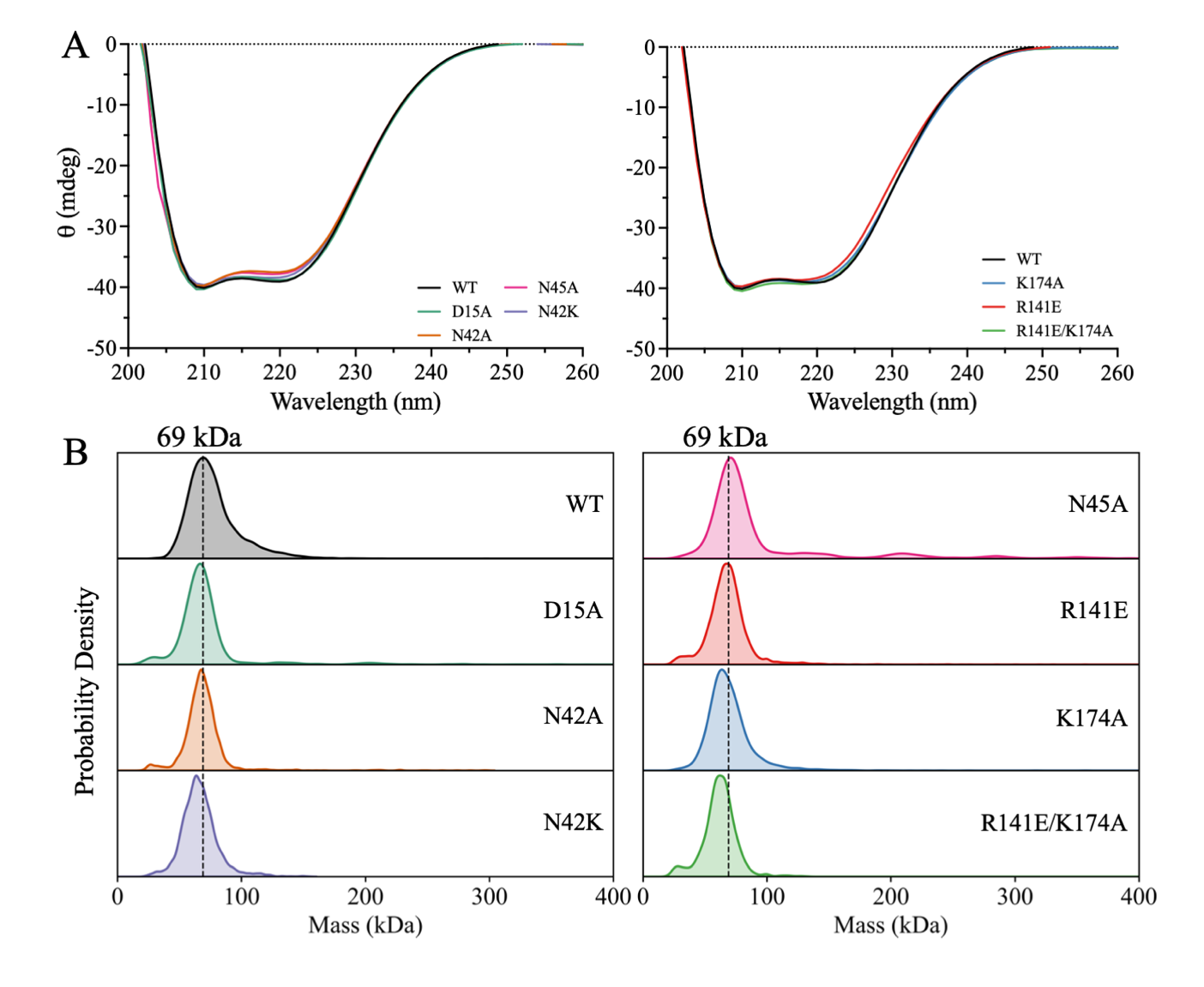


**Figure S2. Structural and oligomeric characterization of KHK-A proteins.** (**A**) Far-UV CD spectra of the KHK-A WT and variants from 200 – 260 nm at 25 °C. All proteins, including WT and mutant KHK-A, exhibited similar far-UV CD spectra with dual ellipticity minima at 208 nm and 222 nm. The spectrum of each protein is the average of five independent measurements. (**B**) The mass distributions (0-400 kDa) of the KHK-A WT and variants were estimated using mass photometry. A vertical dashed line represents the apex of the probability distribution of the WT, with an estimated molecule weight of 69 kDa that is equal to the theoretical value for KHK-A enzyme. Each variant is color-coded and labeled accordingly.


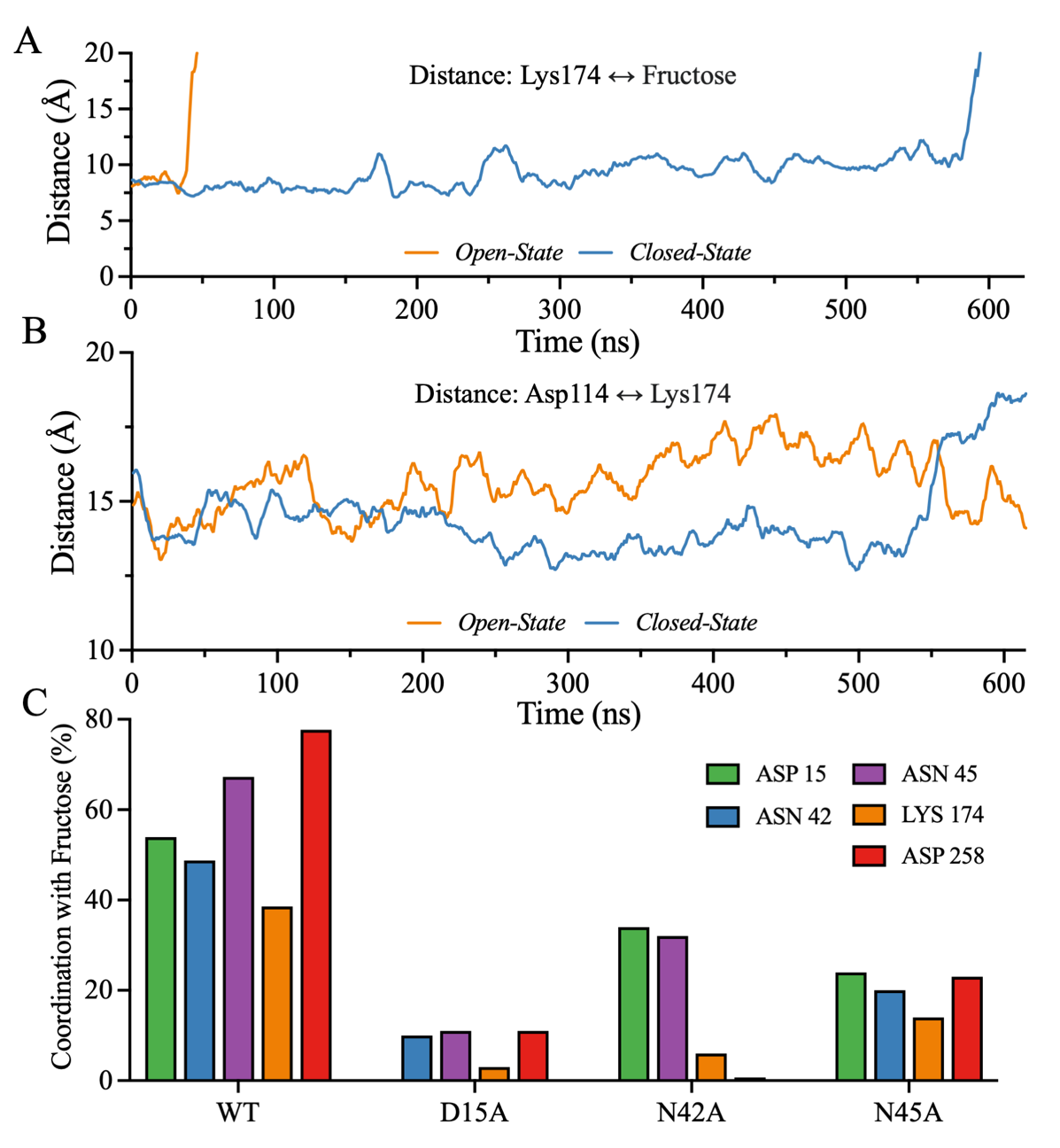


**Figure S3.** **MD simulation analysis of fructose binding to KHK-A.** (**A**) The time dependence distance distribution between the side chain of Lys174 and the hydroxyl of C6 of fructose for the two protomers of KHK-A. Fructose leaves early from one of the protomers at 50 ns compared to the other protomer that binds fructose longer for 600 ns. This suggests that one of the protomers will be present in the open-state while the other is present in the closed-state. (**B**) Fructose coordination percentage for various active site residues in the WT and inactive mutants D15A, N42A, and N45A. Asp258 is the catalytic residue and the WT enzyme showed the highest coordination of fructose with Asp258. All the inactive mutants showed low coordination with fructose, which supports that KHK-A inactivation may result from the inability to bind fructose.


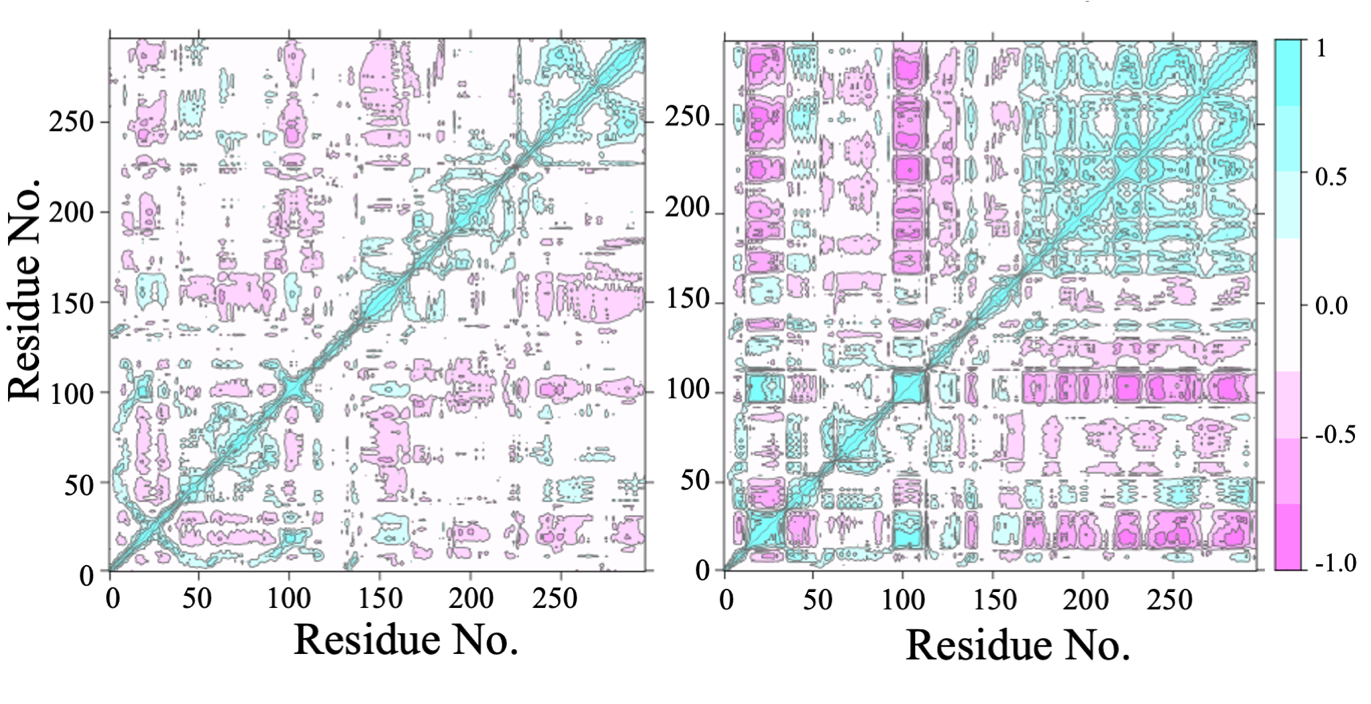


**Fig S4. DCCM plot of the protomer in the open-state and the closed-state**. Positively and negatively correlated motions are represented by cyan and pink, respectively.


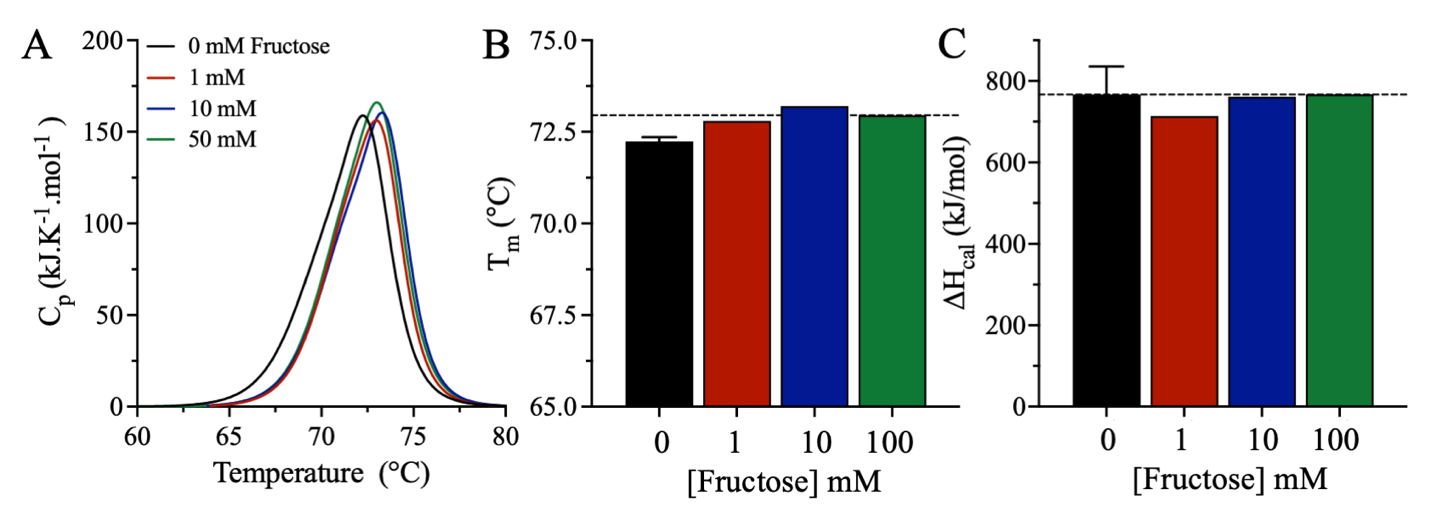


**Figure S5. Unfolding transitions of WT KHK-A at different fructose concentrations.** (**A**) DSC thermograms of WT KHK-A at varying concentrations of fructose (1 mM (blue), 10 mM (red), and 50 mM (black)). Bar plots of (**B**) T_m_ and (**C**) ∆H_cal_.

**Table S1.** Data collection and refinement statistics of KHK-A (PDB ID: 2HLZ).

| **Data Collection** |  |
| --- | --- |
| Space Group: | P1 |
| Cell dimensions: a, b, c (Å) | 66.9, 71.7, 82.7 |
| Angles (°) α, β, γ | 105.06, 107.33, 93.09 |
| Resolution (Å) | 30-1.85 (1.92-1.85) |
| Unique HKLs | 113,793 |
| Completeness(%) | 95.2 (94.6) |
| Rmerge (%) | 7.7 (43.8) |
| Redundancy | 3.8 (3.2) |
|  |  |
| **Refinement** |  |
| Resolution (Å) | 29.7-1.85 |
| No. of reflections (test set) | 113173 (4258) |
| No. of atoms: protein/others | 9045/404 |
| Rwork/Rfree (%) | 21.4/24.9 |
| Average B factors (Å^2^) | 19.3 |
| Protein | 19.3 |
| Water | 21.0 |
| R.m.s. deviations Bond lengths/angles | 0.016/1.382 |
| Ramachandran plot  favored/outliners | 98.6% (1177)/0.08%(1) |

*Values in parentheses are for the highest-resolution shell

**Table S2.** Kinetic parameters of WT and partially active mutants at 37 °C and pH 7.5.

| KHK- A | *V/E_t_* (s^-1^) | *K*_Fru_ (mM) | *K*_ATP_ (mM) | *V*/K_Fru_ *E_t_* (mM^-1^ s^-1^) | *V*/K_ATP_ *E_t_* (mM^-1^ s^-1^) |
| --- | --- | --- | --- | --- | --- |
|  | **Fold Change** | **Fold Change** | **Fold Change** | **Fold Change** | **Fold Change** |
| WT | 1.52 ± 0.2 | 4.4 ± 0.38 | 0.65 ± 0.04 | 0.34 ± 0.01 | 2.31 ± 0.05 |
| R141E | 0.72 ± 0.3  **-2** | 5.92 ± 1.3  **+1.3** | 0.57 ± 0.07 | 0.12 ± 0.01  **- 2.8** | 1.26 ± 0.02  **-1.8** |
| K174A | 0.93 ± 0.1  **-1.6** | 10.65 ± 0.91  **+2.42** | 0.47 ± 0.03  **-1.4** | 0.09 ± 0.01  **-3.9** | 1.99 ± 0.05  **-1.2** |
| R141E/K174A | 0.55 ± 0.1  **-2.7** | 17.18 ± 1.3  **+3.9** | 0.623 ± 0.03 | 0.03 ± 0  **-10.6** | 0.88 ± 0.03  **-2.6** |
